# Sex- and age-dependent neurovascular abnormalities linked to neuroinflammation lead to exacerbated post-ischemic brain injury in Marfan syndrome mice

**DOI:** 10.1016/j.redox.2025.103662

**Published:** 2025-05-07

**Authors:** Gemma Manich, Belén Pérez, Clara Penas, Ana Paula Dantas, Joana Coutinho, Paula Sánchez-Bernadó, Julián García-Aranda, Juan Fraile-Ramos, Núria Benseny-Cases, Beatriz Martín-Mur, Anna Esteve-Codina, Isaac Rodríguez-Rovira, Lydia Giménez-Llort, Gustavo Egea, Francesc Jiménez-Altayó

**Affiliations:** a Department of Morphological Sciences, School of Medicine, Universitat Autònoma de Barcelona, Cerdanyola Del Vallès, Spain; b Institute of Neurosciences, Universitat Autònoma de Barcelona, Cerdanyola Del Vallès, Spain; c Department of Pharmacology, Therapeutics, and Toxicology, School of Medicine, Universitat Autònoma de Barcelona, Cerdanyola Del Vallès, Spain; d Department of Cell Biology, Physiology and Immunology, Universitat Autonoma de Barcelona, Cerdanyola Del Vallès, Spain; e Centro de Investigación Biomédica en Red Sobre Enfermedades Neurodegenerativas (CIBERNED), Instituto de Salud Carlos III, Madrid, Spain; f Red Española de Terapias Avanzadas (RED-TERAV), Instituto de Salud Carlos III, Madrid, Spain; g Department of Biomedical Sciences, School of Medicine and Health Sciences, University of Barcelona, Institut D'Investigacions Biomediques August Pi I Sunyer, Hospital Clinic Cardiovascular Institute, Barcelona, Spain; h Department of Psychiatry and Forensic Medicine, School of Medicine, Universitat Autònoma de Barcelona, Barcelona, Spain; i Unitat de Biofísica. Department of Biochemistry and Molecular Biology, School of Medicine, Universitat Autònoma de Barcelona, Barcelona, Spain; j Centro Nacional de Análisis Genómico (CNAG), Barcelona, Spain; k Universitat de Barcelona, Barcelona, Spain; l Department of Biomedical Sciences, School of Medicine and Health Sciences, University of Barcelona-IDIBAPS, Barcelona, Spain; m Department of Medical Genetics, University of Antwerpen, Antwerpen, Belgium; n Centro de Investigación Biomédica en Red de Enfermedades Cardiovasculares (CIBERCV), Instituto de Salud Carlos III, Madrid, Spain; o Instituto de Investigaciones Biomédicas de Barcelona-Consejo Superior de Investigaciones Científicas (IIBB-CSIC), Institut d'Investigació Biomèdica Sant Pau (IIB SANT PAU), Barcelona, Spain

**Keywords:** Marfan syndrome, fibrillin 1, Neurovascular complications, Ischemic brain injury, Neuroinflammation, Extracellular matrix

## Abstract

Fibrillin 1 gene (*Fbn1*) mutations cause Marfan syndrome (MFS), triggering life-threatening aortic complications and multi-organ effects. MFS is increasingly linked to neurovascular complications, amplified by aortic surgery risks. However, the impact of MFS on the brain remains unclear, including the roles of sex, aging, and their contribution to cerebral injury. This study examines brain alterations and their role in cerebral ischemic injury in an MFS mouse model. RNA-seq analysis of young (3-month-old) and aged (13-month-old) male and female wild-type and MFS (*Fbn1*^*C1041G/+*^) mice revealed disruptions in TGF-β and extracellular matrix (ECM) pathways in MFS brains, most pronounced in young males and aged females with reduced estrogen levels. Inflammatory pathways were upregulated across all MFS mice. Consequently, changes in TGF-β signaling, ECM turnover, redox stress and inflammatory pathways were assessed through RT-qPCR, immunostaining, Western blot, lucigenin chemiluminescence, spectrophotometry, HPLC, and synchrotron radiation-based microspectroscopy, while cerebrovascular properties were assessed by pressure myography and confocal microscopy in the basilar artery. Aged MFS mice showed decreased brain TGF-β1 levels, while dysregulated collagen turnover was only observed in female MFS mice. Despite increased NADPH oxidase activity and redox damage in the corpus callosum of male MFS mice, brain redox stress levels remain largely unchanged. Young female MFS mice exhibited hypertrophic remodeling of the basilar artery. Remarkably, neuroinflammation driven by reactive gliosis increased in MFS mice, regardless of sex and age. To determine the impact on ischemic vulnerability, young mice underwent bilateral common carotid artery occlusion (5 min)/reperfusion (3 days). MFS mice showed greater post-ischemic brain damage, evidenced by worsened behavioral impairments, hippocampal neurodegeneration, and neuroinflammation. This study identifies sex- and age-dependent disruptions in TGF-β1, ECM, and cerebrovascular integrity in MFS mice. Persistent neuroinflammation and increased vulnerability to post-ischemic brain injury suggests that MFS patients, alongside well-documented aortic complications, have an intrinsic predisposition to cerebral damage.

## Highlights

• Marfan mice show altered brain TGF-β and ECM pathways.
• Male Marfan mice show increased redox stress damage in the corpus callosum.
• Young female Marfan mice exhibit basilar artery hypertrophic remodeling.
• Marfan mice show early, persistent neuroinflammation and greater ischemic injury.
• Findings suggest intrinsic predisposition to cerebral damage in Marfan syndrome.

## Graphical abstract

**Figure:**
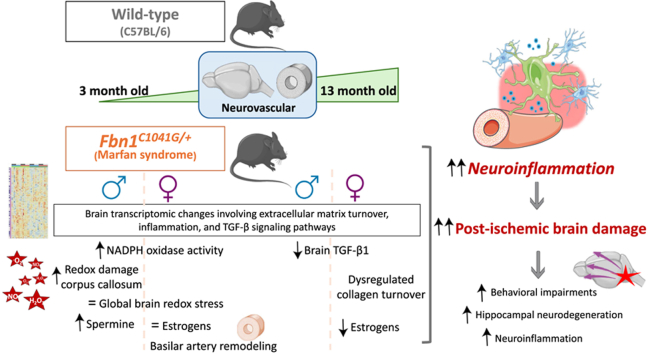

## 1 Introduction

Marfan syndrome (MFS) is a rare genetic disorder of autosomal dominant inheritance with an incidence of 1:5000 individuals and with equal prevalence between sex or ethnic groups. This condition is caused by pathogenic variants in the fibrillin-1 gene (*Fbn1* in mice), a structural glycoprotein that assembles into microfibrils and a basic component of elastic fibers. As a connective tissue disorder, MFS is associated with variable phenotypic features, which include skeletal, ocular, skin, cardiovascular, and pulmonary abnormalities. However, the most life-threatening complication, which manifests more aggressively in men and pregnant women, is early-life aortic dissection, particularly when undiagnosed and pharmacologically untreated [1].

Medical advancements, especially in surgical treatments for aortic aneurysms, have significantly prolonged the life expectancy of patients with MFS [2]. Consequently, people with MFS become more prone to other complications, mainly those related to the neurovascular system [3]. Moreover, prophylactic open thoracic aorta surgery, the most effective current strategy in managing MFS, is linked to a higher risk of neurological complications, such as stroke [1]. Previous retrospective cohort studies have proposed that MFS is linked to an elevated prevalence of intracranial aneurysms [[4], [5], [6], [7]], an assumption that is supported by different clinical case reports, although other studies have not found clear evidence of such a relationship [[8], [9], [10]].

While clinical studies in humans highlight the increased risk of neurovascular complications in MFS patients, preclinical models in mice provide a deeper understanding of the underlying mechanisms driving cerebrovascular vulnerability. *Fbn1* mutation in female apolipoprotein E (ApoE)-deficient mice (*ApoE*^*−/−*^*Fbn1*^*C1041G/+*^) confers susceptibility to brain injury after Western-type diet feeding, evidenced by disturbed cerebral flow, hypoxia, and blood-brain barrier (BBB) permeability [11,12]. Subsequent studies reported redox stress and structural alterations in middle cerebral arteries from *Fbn1*^*C1041G/+*^ mice, associated with dysregulation of transforming growth factor (TGF)-β, NADPH oxidase 4 (Nox4), and matrix metalloproteinase (MMP) 9 expression [13]. Recent studies have reported that 6-month-old male *Fbn1*^*C1041G/+*^ mice showed reduced blood flow velocity in the posterior cerebral artery compared with age-matched controls, while no difference was noted between female mice [14]. Persistent dysregulation of TGF-β signaling, a hallmark characteristic of MFS [15], has been associated with age-related neuroinflammation and various alterations in the brain [16,17]. Moreover, clinically relevant psychiatric and neuropsychological issues have been documented in the literature to be associated with MFS [[18], [19], [20], [21]]. Nonetheless, despite evidence of potential cerebrovascular and brain parenchymal abnormalities in MFS, routine brain monitoring is not currently advised in patients, most likely because of the lack of a thorough characterization of these alterations.

In the present study, we deepen into the effects of *Fbn1* mutation on the brain, utilizing the *Fbn1*^*C1041G/+*^ mouse model of MFS [22]. In addition, we investigated the potential repercussions of bilateral common carotid artery occlusion (BCCAO), a well-recognized method for inducing temporary global cerebral ischemia [23]. Our primary goal was to know whether MFS mice showed baseline brain abnormalities and if these alterations could enhance the risk of cerebral injuries following a transient ischemic episode. Furthermore, we aimed to uncover similarities and differences associated with sex and aging, as well as the underlying mechanisms driving these putative changes.

## 2 Materials and methods

### 2.1 Animals

We used 3-, 7-, and 13-month-old male and female *Fbn1*^*C1041G/+*^ (MFS; *n* = 89) mice, along with their sex- and aged-matched wild-type littermates (WT; *n* = 95). All mice were of the C57BL/6J background strain. The original breeding males were generously provided by Dr. Juan Miguel Redondo (Centro Nacional de Investigaciones Cardiovasculares, Madrid, Spain), and were subsequently backcrossed with WT females obtained from The Jackson Laboratory (Bar Harbor, ME, USA). They were housed in accordance with the institutional guidelines of the Universitat Autònoma de Barcelona. The housing conditions included a constant room temperature of 22 °C, a controlled environment with a 12/12-h light/dark cycle, 60 % humidity, and *ad libitum* access to food and water. All experiments were conducted in compliance with the guidelines established by the legislation on the protection of animals used for scientific purposes in Spain (RD 53/2013) and the European Union (2010/63/EU). The protocols were approved by the ethics committee of the Universitat Autònoma de Barcelona (approval code: CEEAH 5600) and by the Generalitat de Catalunya (approval code: 11,517).

### 2.2 Tissue preparation

Mice (3- and 13-month-old) were deeply anesthetized with isoflurane (5 %; O_2_ and N_2_O, 30:70) and euthanized. Blood samples (1 ml) were collected via intracardial puncture in sodium citrate and centrifuged to separate plasma. The brain was gently dissected and rapidly placed in cold and oxygenated (95 % O_2_ and 5 % CO_2_) Krebs-Henseleit (KH) solution (composition in mM: NaCl 112.0, KCl 4.7, CaCl_2_ 2.5, KH_2_PO_4_ 1.1, MgSO_4_ 1.2, NaHCO_3_ 25.0, and glucose 11.1). The basilar artery was dissected under a surgical microscope and quickly immersed in a cold KH solution and immediately used for either pressure myography, microscopic fluorescence studies of cell nuclei distribution [13], *in situ* oxidative stress evaluation or, after 4 % paraformaldehyde fixation, for immunofluorescence studies. The remaining cerebral arteries were processed for mRNA level analysis by real-time quantitative reverse transcription PCR (RT-qPCR) [13]. Subsequently, brain coronal sections were obtained and processed for RNA-seq and RT-qPCR (striatum-hippocampus, left hemisphere), Western blot (striatum-hippocampus, right hemisphere), high-performance liquid chromatography (HPLC) (prefrontal cortex or midbrain-cerebellum), malondialdehyde (MDA) (prefrontal cortex), and NO (midbrain). The brains of mice (3-month-old) subjected to BCCAO were gently dissected, cut into coronal sections, and processed for immunohistochemistry, immunofluorescence, Fluoro-Jade B staining (hippocampus), HPLC (prefrontal cortex), MDA (prefrontal cortex), and NO (cerebellum).

A separate group of mice, aged 3, 7, and 13 months, were anesthetized during the estrous phase of their estrous cycle, as determined by vaginal smear/cytology. Animals received an intraperitoneal injection of ketamine (80 mg/kg) and xylazine (20 mg/kg) solution at a dosage of 0.01 ml/g body weight. Blood samples (1 ml) were collected via intracardial puncture in sodium citrate and centrifuged to separate plasma. Plasma estrogen concentrations were determined using a commercial ELISA kit (Cayman Chemical, Ann Arbor, MI, USA) and following the manufacturer's instructions. Mice were intracardially perfused with 4 % paraformaldehyde, and their brains were gently dissected, sectioned coronally, and the striatum-hippocampus region was processed for immunohistochemistry, immunofluorescence, and synchrotron radiation-Fourier transform infrared microspectroscopy (μSR-FTIR).

### 2.3 RNA isolation, RT-qPCR, and RNA-seq

Brain tissue and cerebral arteries were homogenized with QIAzol lysis reagent (QIAGEN; Hilden, Germany) and using a BioRuptor (Diagenode; Denville, NJ, USA) with 5 cycles (30 s on and off each). Then, RNA was extracted using a RNeasy Midi Kit (QIAGEN) following the manufacturer's guidelines. RNA was quantified with a spectrophotometer (NanoDrop Technologies; Wilmington, DE, USA) and reverse-transcribed using an Applied Biosystems kit (Thermo Fisher Scientific, Inc; Waltham, MA, USA). Subsequently, the expression of target sequences was quantified by RT-qPCR using the SYBR ® Green QPCR Master Mix (Agilent Technologies; Santa Clara, CA, USA) and the corresponding primers (Table 1) in a C1000 Touch-CFX384 Thermocycler (BioRad; Hercules, CA, USA). Glyceraldehyde-3-phosphate dehydrogenase (GAPDH) was used as a housekeeping gene [24].

**Table 1:** Primer sequences used for real-time quantitative PCR studies.

Primer	Forward (5′- 3′):	Reverse (5′- 3′):
IL-1β	CTTCAAATCTCACAGCAGCACATC	CTTCAAATCTCACAGCAGCACATC
IL-6	AACCACGGCCTTCCCTACTTCA	AACCACGGCCTTCCCTACTTCA
IL-10	AGCCGCCATGAGAGCTAAG	AGCTCCAAGGCACCTGTTC
IL-4	GGCTTTCCTCTTTCCCACTC	AGCCGCCATGAGAGCTAAG
TGF-β1	CCGCAACAACGCCATCTATG	CCCGAATGTCTGACGTATTGAAG
TNFα	AGGCACTCCCCCAAAAGATG	AGGCACTCCCCCAAAAGATG
TNFR2	CAGATCCTCGTGTTGGGATT	CAGATCCTCGTGTTGGGATT
CD68	CCAATTCAGGGTGGAAGAAA	CCAATTCAGGGTGGAAGAAA
iNOS	GGCCAGCCTGTGAGACCTTT	GGCCAGCCTGTGAGACCTTT
Nox1	CCCAGCAGAAGGTCGTGATT	GCTAAAGCCTCGCTTCCTCAT
Nox2	CAGGAACCTCACTTTCCATAAGAT	AACGTTGAAGAGATGTGCAATTGT
Nox4	CCGGACAGTCCTGGCTTATCT	TGCTTTTATCCAACAATCTTCTTGTT
C3	AACAAGCTCTGCCGTGATGA	GCCTGACTTGATGGTCTGCT
S100A10	TGAGAGTGCTCATGGAACGG	AGAAAGCTCTGGAAGCCCAC

IL, interleukin; TGF, transforming growth factor; TNF, tumour necrosis factor; TNFR, TNF receptor; CD, cluster of differentiation; iNOS, inducible nitric oxide synthase; Nox, nicotinamide adenine dinucleotide phosphate oxidase; C3, complement 3; S100A10, S100 calcium-binding protein A10.

RNA-seq reads were mapped against the *Mus musculus* reference genome (GRCm39) using STAR aligner version 2.7.8a [25] with ENCODE parameters. Annotated genes were quantified with RSEM version 1.3.0 [26] with default parameters, using the annotation file from GENCODE version M32. Differential expression analysis was performed with the limma v3.54.2 R package, using TMM normalization. The voom function [27] was used to estimate the mean-variance relationship and to compute observation-level weights. The linear model was fitted with the voom-transformed counts and contrasts were extracted. A gene set enrichment analysis (GSEA) was performed with the R package fgsea v1.12.0 using, for each comparison, the list of pre-ranked genes by the limma moderated t-statistic against the pre-defined mouse REACTOME gene sets. GSEA results were represented by plots generated with ggplot 2.

### 2.4 Western blot

Brain tissue was homogenized using a Polytron homogenizer in RIPA lysis buffer (Thermo Fisher Scientific Inc.) with Halt™ Protease and Phosphatase Inhibitor (Thermo Fisher Scientific Inc.). After 30 min of incubation on ice, samples were centrifuged for 20 min at 14,000×*g* at 4 °C to yield a solubilized preparation. The protein concentration was measured using the BCA protein assay (Thermo Fisher Scientific Inc.). An equal amount of protein from each sample (50 μg) was resolved by SDS-PAGE on a 4–12 % Bis-Tris pre-casted gel (Thermo Fisher Scientific Inc.) and electroblotted onto nitrocellulose membranes. Protein loading control was determined by Ponceau S Staining (Thermo Fisher Scientific Inc.), following manufacturer instructions. Following washes, membranes were incubated overnight at 4 °C in phosphate-buffered saline with 0.1 % (v/v) Tween 20 (PBST) containing 5 % BSA and the specified primary antibodies as follows: rabbit monoclonal anti-Collagen I, 1:1000 (#ab279711, Abcam; Cambridge, UK); goat polyclonal anti-MMP9, 1:1000 (#AF909, R&D Systems; Minneapolis, MN, USA); rabbit monoclonal anti-phospho-SMAD2 1:1000 (#3108, Cell Signaling Technology; Danvers, MA, USA); rabbit monoclonal anti-phospho-Erk 1/2 1:1000 (#9101, Cell Signaling Technology). After several rinses and incubation with specific horseradish peroxidase-labeled specific secondary antibodies diluted in PBST containing 1 % BSA and subsequent additional washes, the chemiluminescent signal was visualized with a LAS4000 imaging system (GE Healthcare; Chicago, IL, USA). Densitometric analyses of western blots were performed using Mac Biophotonic ImageJ® software (U.S. National Institutes of Health; Bethesda, MD, USA), and data were normalized to corresponding values of Ponceau densitometry.

### 2.5 Immunostaining

Free-floating cryostat sections (30 μm) were processed for immunohistochemistry against glial fibrillary acidic protein (GFAP), ionized calcium-binding adapter molecule 1 (Iba-1), and TGF-β1. Briefly, sections were washed with Tris-buffered saline (TBS, pH 7.4) and inhibited for endogenous peroxidase by incubating samples with a solution of 2 % H_2_O_2_ in 70 % methanol for 10 min. Afterwards, sections were incubated for 1 h in blocking solution containing 10 % fetal calf serum and 3 % bovine serum albumin (BSA) in TBS with 1 % Triton X-100 (TBS-T). Then, sections were incubated with rabbit anti-GFAP (1:2000; #Z0334, Agilent Technologies), anti-Iba1 (1:1000; FUJIFILM Wako Pure Chemicals; Osaka, Japan), or TGF-β1 (1:100; #MAB240, R&D Systems; Minneapolis, MN, USA) diluted in the blocking solution, either overnight at 4 °C (or for TGF-β1, 48 h at 4 °C) plus an additional 1 h at room temperature. Negative controls were performed using sections incubated in media lacking the primary antibody, and spleen sections were used as a positive control. After washing three times with TBS-T, sections were incubated at room temperature for 1 h with biotinylated anti-rabbit (1:500; #BA-1000, Vector Laboratories, Inc; Burlingame, CA, USA) secondary antibody diluted in the blocking solution. After 1 h at room temperature in horseradish streptavidin-peroxidase (1:500; #SA-5004, Vector Laboratories, Inc), the final reaction was visualized by incubating the sections with a DAB kit (#SK-4100, Vector Laboratories, Inc) following the manufacturer's instructions. Finally, sections were mounted on gelatin-coated slides, counterstained with toluidine blue, dehydrated in graded alcohols, and, after xylene treatment, coverslipped with DPX. Sections were photographed with a DXM 1200F Nikon (Nikon Inc.; Tokyo, Japan) digital camera attached to a Nikon Eclipse 80i microscope.

Determination of phospho-NRF2 was performed with double immunofluorescence as follows: sections were washed in TBS and incubated in blocking solution. Afterwards, sections were incubated with a rabbit monoclonal antibody against phospho-NRF2 (1:100; #ab76026, Abcam) diluted in blocking solution overnight at 4 °C plus an additional 1 h at room temperature. After washing, sections were then incubated with a donkey anti-rabbit secondary antibody (1:200) conjugated to Cyanine 3 (Jackson ImmunoResearch Laboratories; West Grove, PA, USA) for 1 h at room temperature, and after washes, counterstained with DAPI (1:10,000; Sigma-Aldrich) and covered with Fluoromount mounting medium (Thermo Fisher Scientific Inc.).

Quantitative analysis was performed on sections individually immunolabeled for GFAP, Iba-1, and TGF-β1. Three to 4 animals for each experimental group, containing at least 3 representative brain sections of the areas of interest (cortex, hippocampus, striatum, and/or corpus callosum) were photographed at ×10 magnification. For each marker, the percentage of the area covered by immunolabeling and/or the intensity of the immunoreaction (measured as the mean grey value and expressed in arbitrary units), were quantified using the AnalySIS ® software [28]. The ratio of phospho-NRF2-positive to total nuclei in the hippocampus was quantified using ImageJ software (U.S. National Institutes of Health) in at least four hippocampus per animal.

### 2.6 HPLC

A quantitative measure of superoxide anion levels was performed indirectly by determining brain and plasma concentrations of 2-EOH (Sigma-Aldrich; St. Louis, MO, USA) by HPLC with fluorescence detection, as described [[29], [30], [31]]. 2-EOH was quantified by comparing it with a calibration curve based on the reaction of xanthine-xanthine oxidase [32]. The flow of superoxide anion was increased from xanthine oxidase in the presence of an equal amount of xanthine and a controlled amount of hydroethidine. The calibration curve was constructed by comparing the production of 2-EOH and the ratio of xanthine oxidase activity/hydroethidine concentration (85–605 nU xanthine oxidase/ng hydroethidine). To eliminate the background formation of 2-EOH, sample aliquots were incubated overnight with the superoxide dismutase mimetic Mn(III)tetrakis (1-methyl-4-pyridyl) porphyrin (Sigma-Aldrich), and background values were subtracted from the data.

Brain polyamine concentrations were quantified according to the method of Wang et al. [33] to achieve a fast and optimum separation of the three derivatives: polyamines, volumes, solution, process, and chromatographic conditions were optimized. A total of 100 μL of brain homogenates (1 g/5 ml of RIPA buffer) was analyzed by HPLC after protein precipitation with acetonitrile. The dry residue was dissolved in 100 μL of HPLC water and derivatization with Dns–Cl (5 mg/mL) at pH 10.0 for 45 min was carried out. The mixture from derivatization was adjusted with acetonitrile (Sigma-Aldrich) and 100 μL was injected into the chromatographic system for determination of putrescine, spermidine, and spermine. Commercial standards (Sigma-Aldrich) were used as references for the detection and quantification of the three polyamines. Polyamines were quantified by fluorescence detection with excitation at 340 nm and emission at 510 using a Kromasil 100-5-C18 column (4.0 × 200 mm, Teknokroma Analítica S.A; Sant Cugat del Vallès, Spain).

### 2.7 MDA concentrations

Brain and plasma MDA levels were measured following the method described by Ohkawa et al. (1979) [34]. Brain tissue was homogenized in PBS (0.1 mg/mL). Fifty microliters of brain homogenate or plasma were used for MDA quantification via spectrophotometry at 532 nm.

### 2.8 NADPH oxidase activity

NADPH oxidase activity was measured in brain tissue using the lucigenin-enhanced chemiluminescence technique, as reported [35]. Briefly, brain tissue was homogenized in ice-cold KH solution containing Lucigenin (5 μM) and 100 μl of homogenate was placed in white opaque 96-well plate. Lucigenin-enhanced chemiluminescence was used to determine superoxide production after adding excess NADPH (100 μM). Chemiluminescence was recorded for 3 min at 37 °C using a microplate luminometer (Tecan Multimode Microplate Reader). To assess specificity, the NADPH oxidase inhibitor apocynin (100 μM) was added to separate wells, and its effect on luminescence was analyzed. Background luminescence was subtracted from all readings, and results were normalized to protein concentration, determined using the Bradford assay.

### 2.9 Nitric oxide levels

Brain levels of NO metabolites (nitrites and nitrates) were determined using the colorimetric Griess reaction, as previously described [36]. Brain tissue was homogenized in PBS at 0.1 mg/mL, and 50 μL of the homogenate was taken for NO quantification via spectrophotometric analysis at 540 nm.

### 2.10 Synchrotron radiation-based Fourier transform infrared microspectroscopy (SR-μFTIR)

We used SR-μFTIR to examine lipid oxidation across various brain regions in 9-μm-thick coronal brain sections. The sections were mounted onto polished calcium fluoride optical windows (CAFP20-1; Crystran, UK). Experiments were carried out at the MIRAS beamline at the ALBA synchrotron (Cerdanyola del Vallès, Spain) using a Hyperion 3000 microscope equipped with a ×36 magnification objective coupled to a Vertex 70 spectrometer (Bruker; Billerica, MA, USA) [37]. Spectra were collected in transmission mode at 4 cm^−1^ resolution, 10 × 10 μm aperture, and 128 scans using Opus 7.5 software (Bruker). Measurements ranged from 4000 to 600 cm^−1^ wavenumbers, and zero filling was performed with a fast Fourier transform, obtaining one point every 2 cm^−1^. Background spectra were collected every 10 min in each calcium fluoride window from a clean area. For each region of interest, approximately 50 spectra were acquired with a step size of 30 μm × 30 μm. Spectra exhibiting strong Mie scattering were eliminated. To eliminate the contribution of the baseline and to improve band resolution, the second derivative of the spectra was calculated using a Savitsky-Golay algorithm with a 13-point filter and a polynomial order of three. In this work, we have focused on the study of the asymmetric stretching vibrations of the aliphatic –CH_2_ (∼2852 cm^−1^) and in the C–O (∼1740 cm^−1^) for the analysis of lipid oxidation, and the protein β-folding peak at (1635 cm^−1^) and α-folding (1654 cm^−1^) for indirect measures of protein oxidation and nitration.

### 2.11 Pressure myography

The structural, mechanical, and myogenic properties of the basilar artery were evaluated using pressure myography (model P100; Danish Myo Technology; Aarhus, Denmark) following previously reported protocols [13,38]. In brief, the vessels were positioned on two glass microcannulas and carefully aligned to ensure parallel vessel walls without any stretching. Subsequently, the artery was equilibrated for 45 min at 60 mmHg in KH solution with oxygenation (95 % O_2_ and 5 % CO_2_) at 37 °C. The intraluminal pressure was gradually decreased to 3 mmHg, and a pressure-diameter curve (ranging from 3 to 120 mmHg) was recorded. Internal and external diameters were measured for 3 min at each intraluminal pressure level. Following this, the artery was equilibrated for 30 min at 40 mmHg in gassed, calcium-free (0 Ca^2+^) KH solution (37 °C) with 10 mM EGTA (Sigma-Aldrich), and a second pressure-diameter curve was obtained under passive conditions. The structural, mechanical, and myogenic parameters were analyzed as reported [38].

### 2.12 Three-dimensional confocal microscopy

Fixed (4 % paraformaldehyde), intact, and pressurized (60 mmHg) basilar arteries were stained with Hoechst 33,342 nuclear dye (10 μg/ml; Sigma-Aldrich) for 30 min [13,38]. Briefly, the arteries were mounted on slides with a well made from silicon spacers to avoid artery deformation. Visualization was performed using a confocal system (FV1000, Olympus Iberia; Barcelona, Spain), capturing stacks of serial optical slices (0.4-μm-thick) from the adventitia to the lumen of each artery. Confocal microscopy enabled clear differentiation of the various basilar artery layers stained with Hoechst 33,342, based on the shape and/or orientation of the cell nuclei [39]. At least two sets of images from different regions were captured in each arterial segment. Quantification was performed using MetaMorph Image Analysis software (Molecular Devices; Sunnyvale, CA, USA), following established methods [38].

### 2.13 Basilar artery *in situ* oxidative stress

*In situ* oxidative stress levels were assessed in 14-μm thick basilar artery cross-sections using the oxidative fluorescence dye dihydroethidium (DHE) [31]. In brief, tissue sections were incubated with DHE (2 μM; Sigma-Aldrich), cover-slipped, and kept in a humidified chamber at 37 °C for 30 min, protected from light. To verify fluorescence specificity, the antioxidant Mn(III)tetrakis (1-methyl-4-pyridyl)porphyrin (1.434 mg/ml) was used as a negative control. Images were captured with a laser confocal microscope (40 × ; FV1000, Olympus Iberia, Barcelona, Spain) under consistent acquisition settings across all experimental conditions. Fluorescence intensity was quantified using ImageJ software (U.S. National Institutes of Health) in at least two sections per artery.

### 2.14 Basilar artery immunofluorescence

Frozen transverse sections (14 μm thick) of basilar arteries were stained as previously described [38]. Briefly, sections were incubated for 1 h with a rabbit monoclonal antibody against phospho-NRF2 (1:100; #ab76026, Abcam). After washing, sections were then incubated at 37 °C for 45 min with a donkey anti-rabbit secondary antibody (1:200) conjugated to Cyanine 3 (#711-165-152, Jackson ImmunoResearch Laboratories). The specificity of the immunostaining was confirmed by omitting the primary antibody, which eliminated the fluorescence signal. The ratio of phospho-NRF2-positive nuclei to total nuclei, stained with Hoechst 33,342 (10 μg/mL; Sigma-Aldrich), was quantified using ImageJ software (U.S. National Institutes of Health) in three sections per artery.

### 2.15 Bilateral common carotid artery occlusion (BCCAO) study design

Supplementary Fig. 1 illustrates the experimental design of this study. Systolic blood pressure (SBP) was measured in conscious mice using the tail-cuff plethysmography method (NIPREM 645, Cibertec; Madrid, Spain), as previously described [40]. Measurements were double-blinded and conducted with respect to genotype (Marfan) and sex. Briefly, mice were placed in a dark, warm cage for 10–15 min to enhance pulse detectability, followed by several cycles of inflation/deflation. The animals were gradually habituated over three consecutive days before SBP measurements were systematically taken between 10:00 a.m. and 12:00 p.m., both one day before and after BCCAO, and then again three days after BCCAO (before euthanasia), as outlined in Supplementary Fig. 1. SBP values for each mouse were determined from the mean value obtained after four consecutive measurements, following the habituation period. Body weight was measured one day before and after BCCAO, as well as before euthanasia. Behavioral tests were systematically performed between 10:00 a.m. and 12:00 p.m. on day 2 post-BCCAO. After euthanasia, neurodegeneration was measured by Fluoro-Jade B staining and brain inflammation by GFAP and Iba-1 immunohistochemistry.

### 2.16 Induction of BCCAO

Transient global cerebral ischemia was induced using the BCCAO technique as previously described [41] with some adjustments. Briefly, the mice were anesthetized with 3 % isoflurane in a mixture of N_2_O:O_2_ (70:30). Rectal temperature was carefully monitored during surgery and maintained at approximately 37.5 °C using a thermal blanket. Both common carotid arteries were closed using surgical clamps. Preliminary experiments were conducted to determine the optimal occlusion duration, ensuring sufficient hypoxic brain damage without causing unacceptable mortality rates. The animals underwent a 5-min occlusion followed by three days of reperfusion. At the end of occlusion, the clamps were removed, and the arteries were visually inspected to confirm reperfusion. The neck incision was then closed with 3-0 non-absorbable silk sutures. For analgesia, 0.01 mg/kg of buprenorphine was administered (*s.c.*). The mice were allowed to recover for three days (reperfusion), after which they were euthanized by decapitation under deep anesthesia (4 % isoflurane; N_2_O:O_2_ 70:30). The study included four experimental groups: male WT (*n* = 14), male MFS (*n* = 12), female WT (*n* = 10), and female MFS (*n* = 11). Investigators were unaware of genotype and sex during and after surgical procedures. Surgery in female mice was conducted to coincide with the estrus phase to reduce variability in experimental outcomes linked to estrogen levels and hormonal fluctuations.

### 2.17 Behavioral tests

Neophobia was evaluated using the corner test (CT) for 30 s [42]. Each animal was placed individually in the center of a clean standard home cage filled with wood-shaving bedding. The recorded parameters included the number of corners visited, latency to the first rearing, and total number of rearings. Following the CT, mice were transferred to the center of an open field (OF; metalwork, white box, 42 × 38 × 15 cm) and observed for 5 min. Behavioral events, such as freezing duration, latency to leave the central square, entry into the peripheral ring, and self-grooming duration were recorded according to a predefined ethogram. Locomotor activities, both horizontal (crossings of 10 × 10 cm squares) and vertical (rearings with wall support), were also quantified. Additionally, defecation and urination were monitored during the tests.

### 2.18 Fluoro-Jade B staining

To detect degenerating neurons, 30 μm cryostat sections of BCAO were stained with Fluoro-Jade B following the protocol outlined by Schmued and Hopkins [43]. In brief, free-floating sections were mounted and air-dried overnight. After dehydration and rehydration in graded ethanol, sections were rinsed in water and oxidized with 0.06 % potassium permanganate (KMnO_4_ in water) for 15 min. Subsequently, sections were rinsed with distilled water and immersed in 0.0004 % Fluoro-Jade B (Histo-Chem Inc.; Jefferson, AR, USA) solution containing 1 % glacial acetic acid for 20 min. After rinsing, slides were air-dried, cleared in xylene, and coverslipped in DPX. Sections were photographed with a DXM 1200F Nikon digital camera attached to a Nikon Eclipse 80i microscope and green fluorescence was detected. For neuronal cell death counting, Fluoro-Jade-B-positive cells were counted in at least four dentate gyrus per animal, and upon determination of the dentate gyrus area using ImageJ® software (U.S. National Institutes of Health), and the number of Fluoro-Jade -B-positive cell/mm^2^ was obtained.

### 2.19 Statistical analysis

Results are presented as mean ± standard error of the mean (SEM), except in Fig. 6, where they are expressed as median [Q1; Q3], from the number of animals (*n*) specified in each table/figure. Before selecting appropriate tests, data distribution was systematically evaluated. Single-factor analysis utilized the unpaired Student's *t*-test for comparing two means. Two-factor analysis involved repeated or regular measures of two-way ANOVA, and three-factor analysis involved regular measures of three-way ANOVA, with Tukey's or Bonferroni's post-hoc test for grouped analyses. Statistical tests employed are indicated in each table/figure where differences exist. Fisher's exact test was used to compare survival rates between groups due to the small sample sizes. Correlations among functional variables, namely, behavioral phenotype, physical status (pre- and post-operation weight), and vascular system (systolic blood pressure pre- and post-operation) were assessed using Pearson's test, initially for the entire population and then segregated by genotype. The data are displayed as heat maps with correlation coefficients. Functional correlations were analyzed using RStudio April 1, 1103 software. Data analysis was performed using GraphPad Prism® version 8.0 and 9.0 (GraphPad Software Inc.; San Diego, CA, USA). Statistical significance was considered at *p* < 0.05.

## 3 Results

### 3.1 Brain transcriptomic analysis shows ECM turnover, inflammation, and TGF-β signaling pathways changes in both young male and aged female MFS mice

**Fig. 1:**
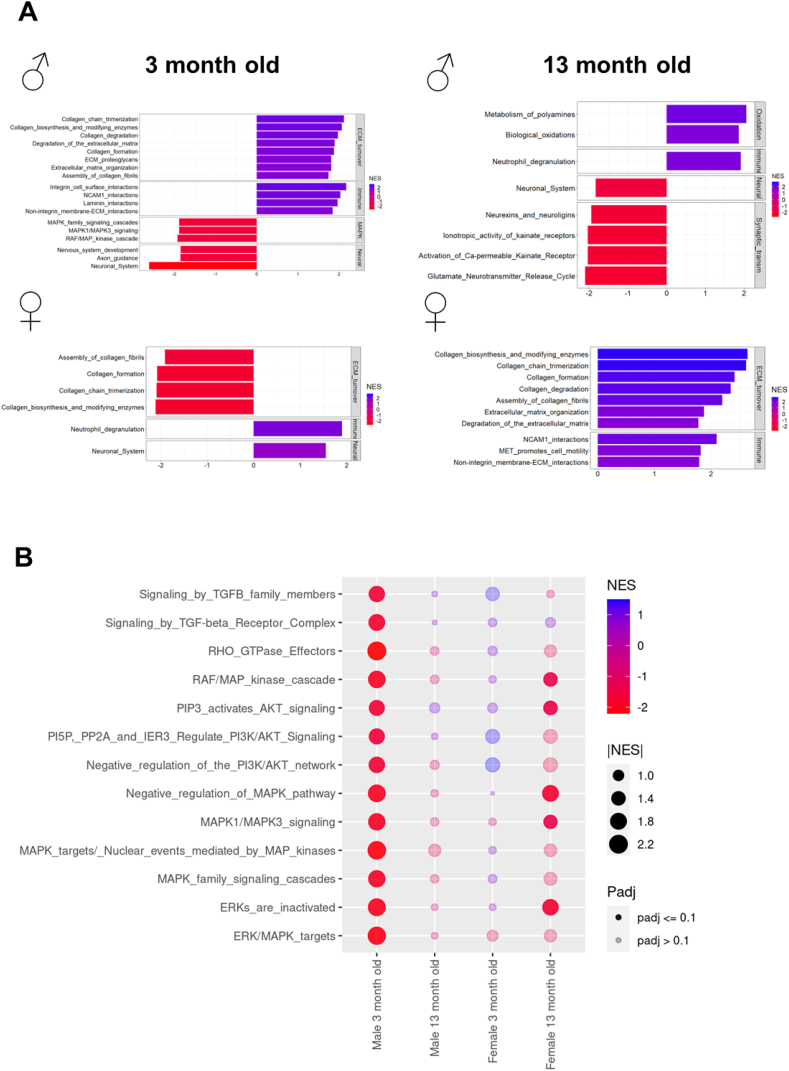
Gene set enrichment analysis (GSEA) in brains from 3- and 13-month-old male and female wild-type (WT) and Marfan syndrome (MFS) mice. (A) Bar plots showing selected statistically significant REACTOME pathways. GSEA normalized enrichment scores (NES) are represented on the x-axis and in a color gradient. (B) Dot plot of combined GSEA results illustrating selected REACTOME TGF-β-related pathways. The color gradient indicates the negative to positive NES change, dot size illustrates absolute NES values, and dot transparency indicates whether the result has an adjusted *p*-value (padj) below or equal 0.1. *N* = 4 per experimental group.

RNA-seq-based transcriptomics was conducted in the brains of young and aged (3- and 13-month-old, respectively) male and female WT and MFS mice. Notably, no individual gene showed significant differential expression between WT and MFS mice. Therefore, we performed a gene set enrichment analysis following differential mRNA expression examination (Fig. 1A). In 3-month-old mouse brains, several pathways related to ECM turnover were significantly upregulated in MFS compared with WT male mice, while some of these same pathways were downregulated in age-matched female MFS mice. However, in 13-month-old mouse brains, collagen turnover pathways were no longer upregulated in MFS compared to WT male mice, while they were markedly upregulated in aged female MFS mice. Of note, inflammation pathways such as adhesion molecule interactions or neutrophil degranulation were overexpressed in MFS mice of both sexes and all ages (Fig. 1A). To further illustrate these findings, Supplementary Fig. 2A presents a heat map depicting the log-normalized expression levels of genes involved in ECM turnover and inflammation pathways. This figure highlights the increased expression of genes related to ECM turnover and inflammation in young male MFS versus WT mice.

Dysregulation of TGF-β signaling is a well-established hallmark of MFS pathology [15]. Given that some TGF-β signaling-related pathways were downregulated in 3-month-old male MFS mice (Fig. 1A), we next compared the enrichment of diverse TGF-β-associated pathways in all experimental groups (Fig. 1B). Consistently, results showed a decreased enrichment in several TGF-β signaling-related pathways in 3-month-old MFS *versus* WT male mice, which disappeared in 13-month-old animals. However, young female MFS mice showed no significant alterations in TGF-β signaling pathways compared to WT mice. Unlike male MFS mice, female MFS showed downregulation of some TGF-β signaling pathways at 13 months of age. Supplementary Fig. 2B presents a heat map of log-normalized gene expression levels, illustrating the progression of TGF-β signaling pathway expression across different experimental groups. Collectively, results indicate that the brains of MFS mice exhibit age- and sex-related changes in gene expression, particularly those involving ECM turnover, inflammation, and TGF-β signaling pathways. These changes follow a similar pattern in young male and aged female MFS mice.

### 3.2 Sex-specific dysregulation of the TGF-β1 pathway and ECM turnover in MFS mouse brains

**Fig. 2:**
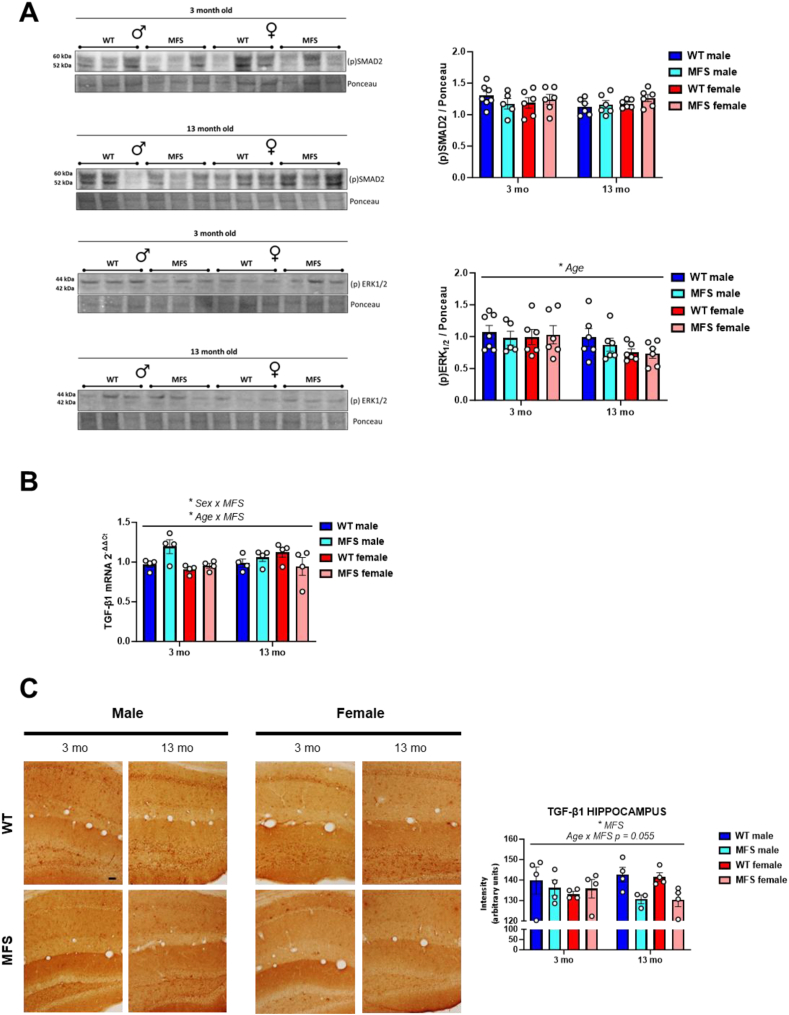
Expression of some representative transforming growth factor (TGF)-β signaling proteins and genes in brains from 3- and 13-month-old male and female wild-type (WT) and Marfan syndrome (MFS) mice. (A) Western blot analysis for phosphorylated SMAD2 (pSMAD2; above) and phosphorylated ERK1/2 (pERK 1/2; below) protein expression in the brain. Bar graphs show the results of densitometric analyses from pooled data. (B) Brain TGF-β1 mRNA expression levels. (C) Representative images of TGF-β1 immunostainings in brain hippocampus. Bar graphs show the results of the intensity of TGF-β1 immunostaining in brain hippocampus obtained by densitometry. Scale bar, 50 μm. Results are the mean ± SEM with each data point representing an animal. ∗*p* < 0.05 by three-way ANOVA with Tukey's post-hoc test.

Given the central role of TGF-β signaling in MFS pathology and the transcriptomic alterations observed, we next examined key components of this pathway in the brain (Fig. 2). Despite brain transcriptomic analyses showing MFS-associated changes in TGF-β signaling pathways (Fig. 1B) and a significant age-related decrease in phospho-ERK1/2 levels (Fig. 2A), phospho-SMAD2 and phospho-ERK1/2 protein levels remained unchanged across genotypes and sexes (Fig. 2A). TGF-β1 transcripts only exhibited interactions between sex and genotype and age and genotype (Fig. 2B). Interestingly, TGF-β1 expression in the hippocampus was statistically lower in both aged female and male MFS mice compared to WT animals (Fig. 2C).

**Fig. 3:**
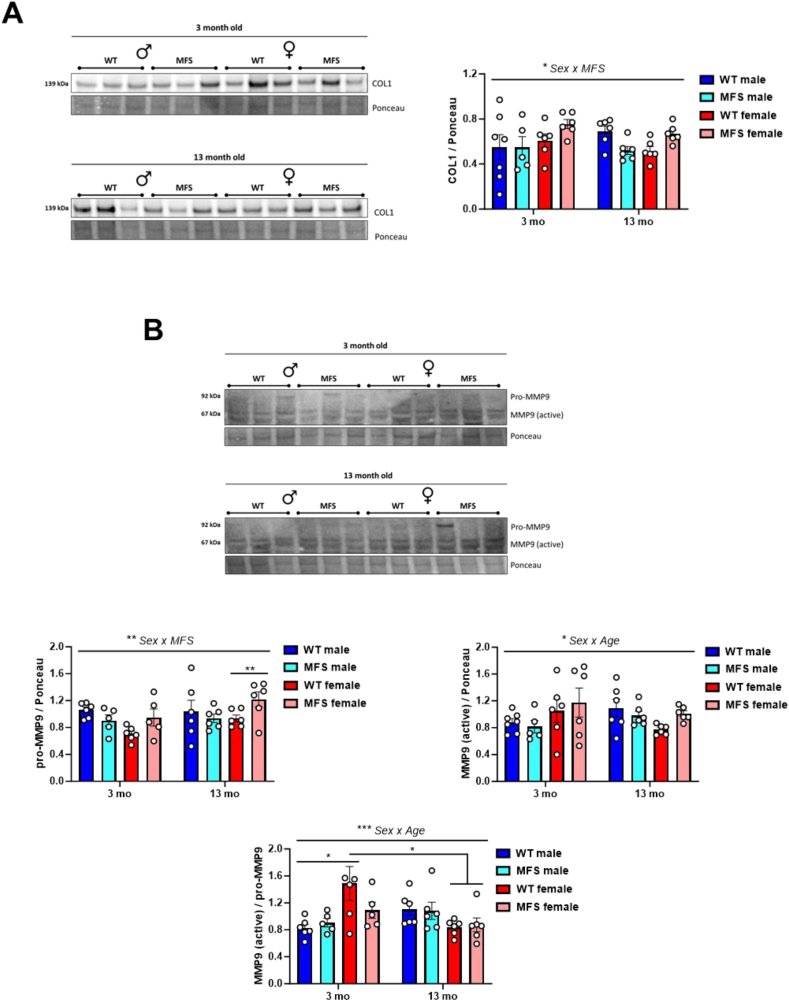
Expression of some representative ECM turnover proteins in brains from 3- and 13-month-old male and female wild-type (WT) and Marfan syndrome (MFS) mice. (A) Western blot analysis for collagen I (COL-1) and (B) pro-matrix metalloproteinase (MMP) 9 (above) and active MMP9 (below) protein expression. Bar graphs show the results of densitometric analyses from pooled data. Results are the mean ± SEM with each data point representing an animal. ∗*p* < 0.05, ∗∗*p* < 0.01, ∗∗∗*p* < 0.001 by three-way ANOVA with Tukey's post-hoc test.

Next, we investigated whether the transcriptomic changes identified in brain ECM turnover pathways were reflected at the protein level. To this end, we first analyzed protein expression levels of collagen I, pro-MMP9, and active MMP9 (Fig. 3). We detected a significant sex-by-genotype interaction in collagen I levels, suggesting that there is a sex-specific dysregulation of brain collagen expression in MFS, independent of age, with increased levels in MFS females (Fig. 3A). Similarly, pro-MMP9 levels also showed a significant sex-by-genotype interaction (Fig. 3B). Notably, 13-month-old female MFS mice showed increased pro-MMP9 expression without changes in the activation ratio (Fig. 3B). This, together with slightly higher collagen I levels and markedly upregulated ECM turnover pathways in the transcriptomic analysis (Fig. 1A), suggests a progression toward potential fibrosis in this group.

Overall, these findings indicate different patterns of TGF-β1 pathway regulation in male and female MFS mice over time. In males, the lower enrichment of TGF-β1 transcriptomic pathways at 3 months, despite unchanged TGF-β1 levels and signaling, suggests early alterations in the pathway. By 13 months, brain TGF-β1 levels are reduced and no changes in pathway enrichment occur. In contrast, female MFS mice exhibit only a slight decrease in the enrichment of TGF-β1 transcriptomic pathways at 13 months, along with reduced brain TGF-β1 levels, which are associated with late-stage dysregulation of matrix turnover.

### 3.3 Redox stress in MFS brains: compensatory antioxidant responses in male and female mice

**Fig. 4:**
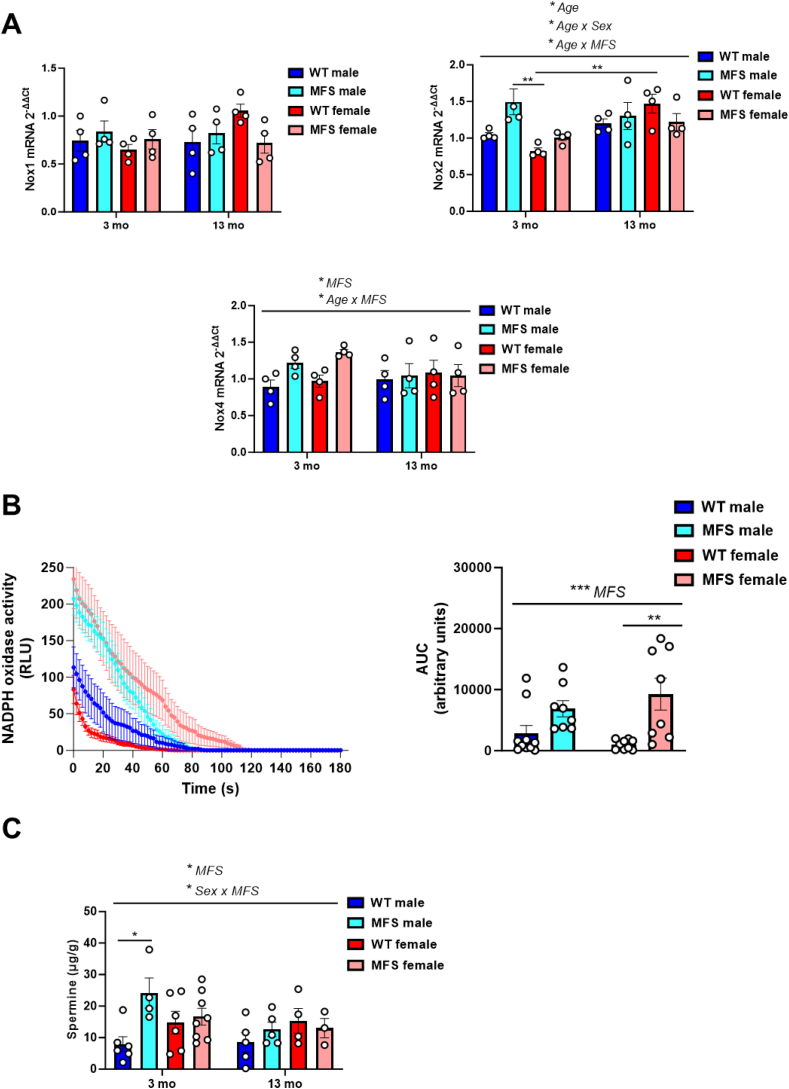
Levels and activity of some representative redox markers measured in brains from 3- and 13-month-old male and female wild-type (WT) and Marfan syndrome (MFS) mice. (A) NADPH oxidase isoforms Nox1, Nox2, and Nox4 mRNA expression levels. ∗*p* < 0.05 by three-way ANOVA with Tukey's post-hoc test. (B) Representative chemiluminescence tracing (left) and the areas under the curve (AUC) of NADPH-dependent O_2_^−^ production in brains from 3-month-old male and female WT and MFS mice. RLU, relative light units. ∗∗*p* < 0.01, ∗∗∗*p* < 0.001 by two-way ANOVA with Tukey's post-hoc test. (C) Spermine concentrations measured by HPLC. Results are the mean ± SEM. Data points represent the number of animals. ∗*p* < 0.05 by three-way ANOVA with Tukey's post-hoc test.

Previous studies have demonstrated a correlation between MFS aortic disease and increased redox stress [[44], [45], [46]]. Therefore, we investigated whether redox stress is similarly elevated in MFS mouse brains. We first examined the mRNA expression of the NADPH oxidases Nox1, Nox2, and Nox4 (Fig. 4A). While Nox1 expression remained unchanged across genotypes, sexes, and ages, Nox2 showed an age-related upregulation and slightly higher levels in young MFS mice. In addition, MFS mice, particularly young ones, displayed higher Nox4 mRNA expression compared to WT mice. To determine whether these transcriptional changes resulted in increased enzymatic activity, we measured NADPH oxidase activity in the brains of 3-month-old mice. The results showed higher activity in MFS mice, with statistical significance observed only in females (Fig. 4B). The addition of apocynin led to a significant reduction in lucigenin-induced chemiluminescence, confirming the specificity of the signal (results not shown). We then measured superoxide anion production in the brain and plasma by analyzing 2-EOH levels using HPLC, yet no significant changes were observed (Supplementary Fig. 3A). Next, we evaluated brain and circulating MDA levels, a marker of lipid peroxidation and, again, no significant differences were detected between groups (Supplementary Fig. 3B). We also explored brain NO levels, given that increased NO-derived inducible NO synthase (iNOS) activity has been implicated in MFS aortic disease [44]. Again, no changes were detected (Supplementary Fig. 3C). Finally, we investigated endogenous polyamines, putrescine, spermine, and spermidine, which are known for their protective antioxidant roles [47]. Notably, 3-month-old male MFS mice exhibited a significant, sex-specific increase in spermine levels compared to WT mice (Fig. 4C; Supplementary Fig. 4A), whereas putrescine and spermidine levels remained unchanged in both sexes (Supplementary Fig. 4A and 4B).

**Fig. 5:**
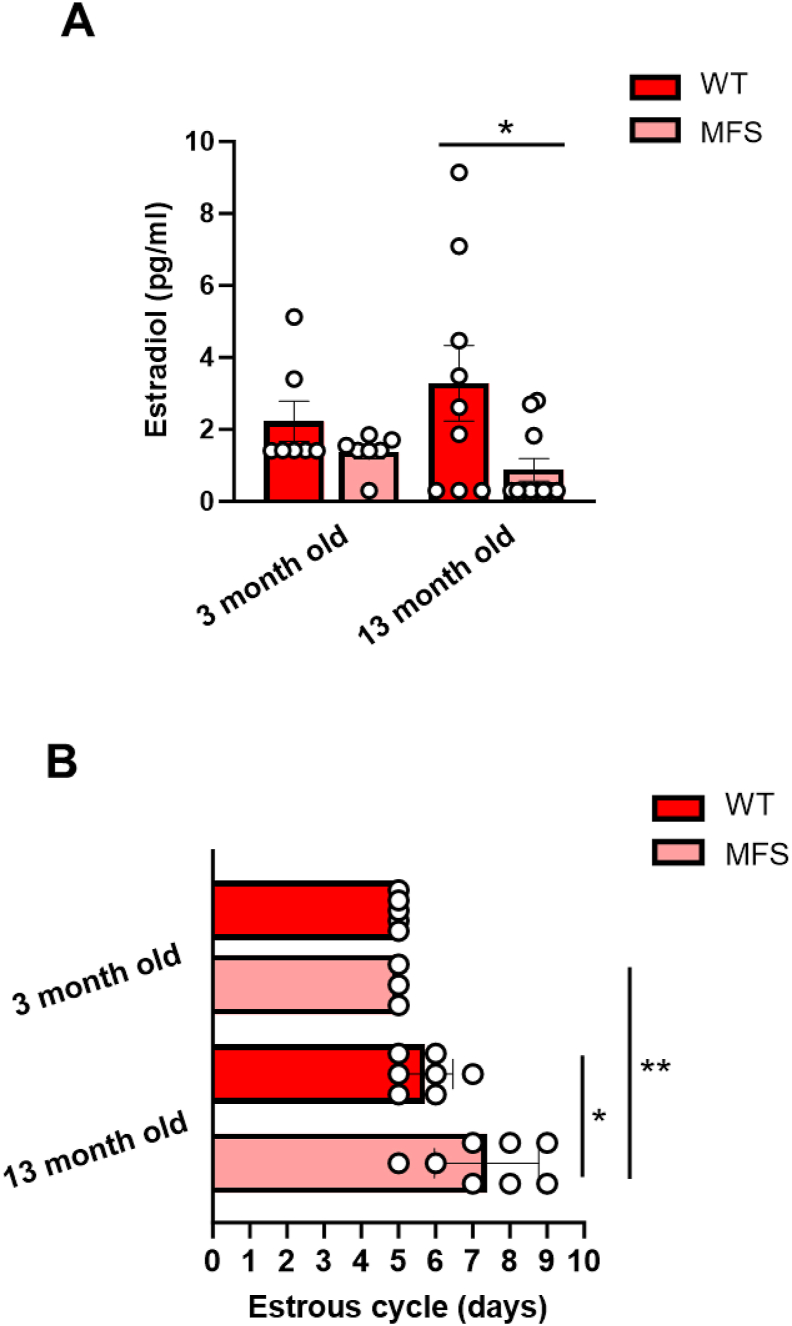
Blood estradiol levels and estrus cycle duration in 3- and 13-month-old female wild-type (WT) and Marfan syndrome (MFS) mice. (A) Estradiol concentrations in plasma measured by spectrophotometry. (B) Duration of the estrous cycle. Results are the mean ± SEM with each data point representing an animal. ∗*p* < 0.05, ∗∗*p* < 0.01 by two-way ANOVA with Tukey's post-hoc test.

The minor brain alterations observed in the transcriptomic, ECM turnover, and redox stress analyses of young female MFS mice contrast with the elevated NADPH oxidase activity and more pronounced alterations observed in aged animals. This observation led us to investigate the role of estrogens in the origin of these notable differences. To this aim, we measured blood levels of estradiol (Fig. 5A) and the duration of the estrus cycle (Fig. 5B). Interestingly, we found comparable blood levels of estradiol and an equal duration of the estrus cycle in young female MFS mice. However, aged MFS animals showed a significant reduction in estradiol levels and a longer estrus cycle. These results suggest that estrogens play a role in brain protection in young female MFS mice, while this effect is reduced in older ones.

**Fig. 6:**
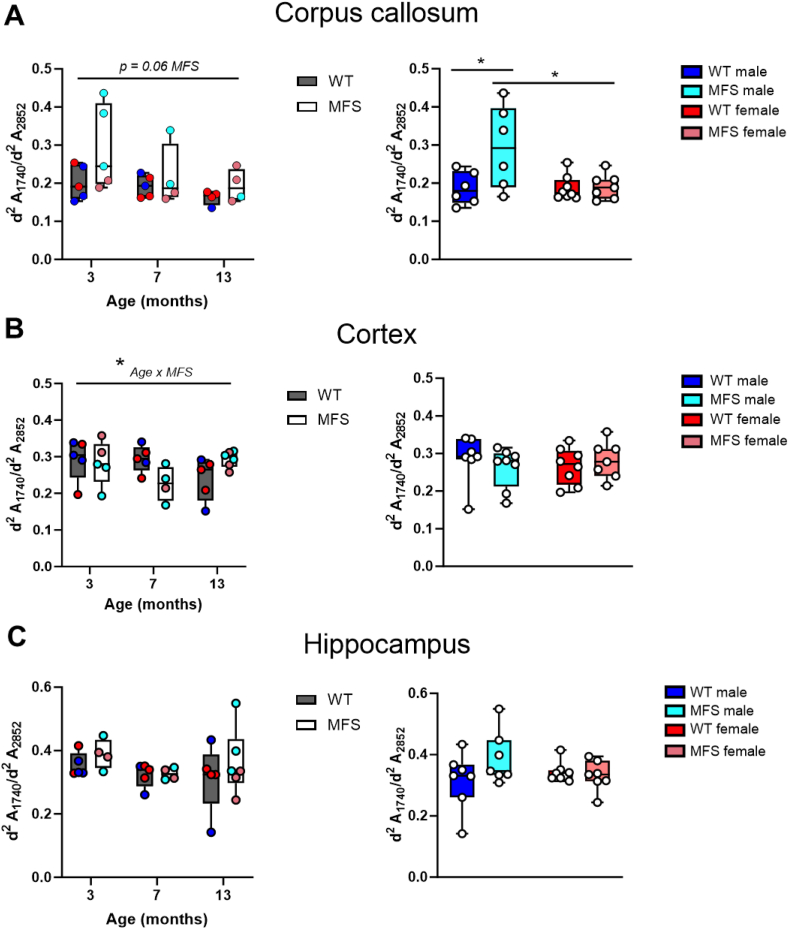
Representation of the aldehyde band (C═O) in the infrared spectra, normalized by the CH_2_ band (estimation of the total lipid content) as a measure of lipid oxidation using μSR-FTIR. The intensity of the bands was measured from the second derivative (d_2_A) of the spectra in (A) corpus callosum, (B) cortex, and (C) hippocampus from 3-, 7-, and 13-month-old male and female wild-type (WT) and Marfan syndrome (MFS) mice. Different colors identify male and female WT and MFS groups. On the right, data from all ages are combined and separated by sex and genotype (MFS). Results are median [Q1; Q3] with each data point representing an animal. ∗*p* < 0.05 by two-way ANOVA with Tukey's post-hoc test.

Overall, despite we observed increased *Nox* gene expression and elevated NADPH oxidase activity in the brains of MFS mice, these changes did not result in widespread redox stress. Notably, the elevated spermine levels in young male MFS mice and the preserved circulating estrogen levels in young female MFS mice suggest the presence of compensatory antioxidant mechanisms.

### 3.4 Region-specific alterations in redox stress damage in MFS mouse brains

To identify brain regions potentially more vulnerable to redox stress, we examined using μSR-FTIR the lipid oxidation and β/α protein folding ratio as indicators of oxidative damage, which could also be linked to nitrosative stress. We measured lipid oxidation by calculating the ratio of the peak of aldehydes at 1740 cm^−1^, normalized by the CH_2_ (2852 cm^−1^) band as a reference for total lipids (A_1740_/A_2852_) [48] (Fig. 6). The lipid oxidation analysis revealed a near-significant increase (*p* = 0.06) in the corpus callosum of MFS mice compared to WT mice (Fig. 6A). Although no overall changes were detected in the cortex (Fig. 6B) and hippocampus (Fig. 6C), a significant interaction between age and genotype was observed in the cortex (Fig. 6B). A more detailed analysis indicated that the increased lipid oxidation in the corpus callosum of MFS mice was primarily attributed to males (Fig. 6A). These alterations occurred despite increased total lipid (CH_2_, A_2852_) content in MFS mice (results not shown). We also assessed β/α protein folding by calculating the ratio of the β-folding peak at 1635 cm^−1^, normalized to the α-folding band at 1654 cm^−1^ [48] (Supplementary Fig. 5). The analysis showed increased β/α protein folding in the corpus callosum of MFS mice, particularly in males, suggesting a potential association with oxidative and nitrosative protein modifications in this cerebral region. These findings suggest that MFS mouse brains exhibit signs of elevated redox stress in the corpus callosum compared with WT mice, with a more pronounced effect in males than in females.

Immunofluorescence analysis of phospho-NRF2 in the hippocampus showed no differences between MFS and WT mice, regardless of sex or age (Supplementary Fig. 6), although phospho-NRF2 tended to be reduced with age, independently of genotype. Analysis in the corpus callosum could not be performed due to high variability in signaling.

Together, these findings indicate that, although widespread brain redox stress is absent, MFS mice, particularly males, exhibit region-specific increases in redox damage in the corpus callosum. These results suggest that certain brain areas are more susceptible to redox damage.

### 3.5 Male and female MFS mice indistinctly show increased neuroinflammation

**Fig. 7:**
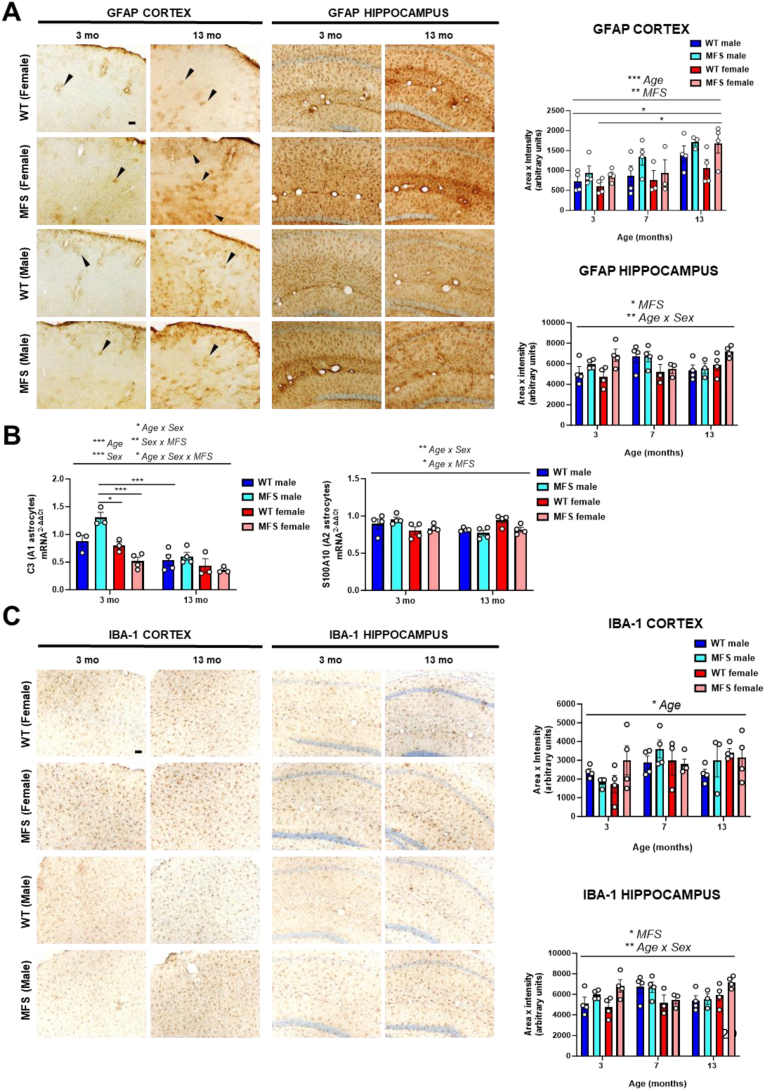
Glial reactivity in 3-, 7-, and 13-month-old female and male wild-type (WT) and Marfan syndrome (MFS) mice. (A) Representative images of astrocytic GFAP immunostainings of 3 and 13-month-old female and male WT and MFS mice in brain cortex and hippocampus. Bar graphs show the results of GFAP immunostaining obtained by densitometry in 3-, 7-, and 13-month-old female and male WT and MFS mice. Representative GFAP-positive astrocytes are indicated with arrowheads in the cortex. Scale bar, 50 μm. (B) C3 and S100A mRNA expression levels. (C) Representative images of microglial Iba-1 immunostaining of 3 and 13-month-old female and male WT and MFS mice in brain cortex and hippocampus. Bar graphs show the results of Iba-1 immunostaining obtained by densitometry in 3-, 7-, and 13-month-old female and male WT and MFS mice. Scale bar, 50 μm. Results are the mean ± SEM. Data points represent the number of animals. ∗*p* < 0.05, ∗∗*p* < 0.01, ∗∗∗*p* < 0.001 by three-way ANOVA with Tukey's post-hoc test.

Considering the enrichment of inflammatory pathways identified by the transcriptomic analysis of brains from MFS mice (Fig. 1A), coupled with the documented impact of TGF-β on neuroinflammation [16,17], we next examined potential neuroinflammation markers in MFS mouse brains compared to WT controls (Fig. 7 and Supplementary Fig. 7). Assessment of astrogliosis revealed baseline GFAP staining in all brain areas analyzed in young WT and MFS mice, which notably increased in the cortex and striatum with age and MFS and in the hippocampus and corpus callosum with MFS (Fig. 7A and Supplementary Fig. 7A, respectively). Transcriptional analysis of C3 and S100A10 (markers for A1 and A2 astrocytes, respectively) revealed elevated C3 mRNA levels in young male MFS mice, while S100A10 levels remained unchanged (Fig. 7B). We also assessed microglial activation through Iba-1 immunostaining. Age-related increases in Iba-1 levels were observed in the cortex (Fig. 7C). Notably, Iba-1 expression was significantly elevated in the hippocampus of MFS mice (Fig. 7C), while levels remained comparable in the striatum and corpus callosum (Supplementary Fig. 7B). Together, these results indicate widespread astrogliosis and region-specific microgliosis in MFS mice.

In addition, we examined the brain mRNA expression levels of several pro- and anti-inflammatory factors (Table 2). Among the factors analyzed, we observed an age-related effect: increased expression of IL-1β, IL-6, TNFα, and CD68, along with decreased expression of TNF receptor 2 and iNOS. We found a genotype effect, with an increased IL-6 mRNA expression in MFS mice. When focusing on specific groups, we observed elevated IL-1β and CD68 mRNA levels in aged female mice regardless of genotype. Notably, IL-6 and IL-10 mRNA expression were higher in 13-months-old male MFS mice compared to WT controls. In conclusion, our findings reveal age-, sex-, and genotype-dependent changes in the expression of key inflammatory factors. Notably, aged females showed elevated levels of IL-1β and CD68, irrespective of genotype, while older male MFS mice showed increased IL-6 and IL-10 in their brains.

**Table 2:** Analysis of pro- and anti-inflammatory factor mRNA expression (2^−ΔΔCt^) in brains from male and female wild-type (WT) and Marfan syndrome (MFS) mice.

	3 MONTH OLD				13 MONTH OLD			
	WT male (*n* = 3–4)	MFS male (*n* = 3–4)	WT female (*n* = 4)	MFS female (*n* = 2–4)	WT male (*n* = 3–4)	MFS male (*n* = 3–4)	WT female (*n* = 4)	MFS female (*n* = 3–4)
**Pro-inflammatory**								
IL-1β	0.93 ± 0.19	0.92 ± 0.05	0.61 ± 0.05	0.60 ± 0.1	0.97 ± 0.05	1.44 ± 0.08	1.55 ± 0.14**^#/+++^**	1.15 ± 0.08^+^
IL-6	0.97 ± 0.02	0.91 ± 0.07	0.83 ± 0.02	1.12 ± 0.01	1.13 ± 0.05	1.85 ± 0.34**∗^/++^**	1.20 ± 0.16	1.36 ± 0.11
TNFα	1.29 ± 0.13	1.30 ± 0.33	0.94 ± 0.05	0.89 ± 0.15	1.56 ± 0.27	2.07 ± 0.48	1.79 ± 0.28	1.70 ± 0.19
TNF receptor 2	1.06 ± 0.06	1.07 ± 0.14	1.05 ± 0.15	0.99 ± 0.04	0.72 ± 0.03	0.75 ± 0.08	0.82 ± 0.12	0.73 ± 0.04
CD68	0.89 ± 0.05	0.86 ± 0.03	0.75 ± 0.05	0.75 ± 0.05	0.89 ± 0.02	0.93 ± 0.03	1.03 ± 0.04**^+++^**	0.93 ± 0.03^+^
iNOS	0.83 ± 0.10	0.94 ± 0.08	0.82 ± 0.06	0.80 ± 0.09	0.70 ± 0.07	0.74 ± 0.05	0.67 ± 0.08	0.69 ± 0.05
**Anti-inflammatory**								
IL-10	1.22 ± 0.11	1.28 ± 0.13	1.27 ± 0.23	0.68 ± 0.09	1.04 ± 0.09	4.04 ± 1.68**∗**	1.57 ± 0.24	1.30 ± 0.03
IL-4	0.56 ± 0.15	0.74 ± 0.09	0.73 ± 0.15	0.65 ± 0.08	0.64 ± 0.14	0.88 ± 0.14	0.64 ± 0.08	0.72 ± 0.06

IL, interleukin; TNF, tumour necrosis factor; CD, cluster of differentiation; iNOS, inducible nitric oxide synthase. Results are the mean ± SEM.

***∗****p* < 0.05 *versus* WT within the same age.

**^#^***p* < 0.05 *versus* same group and age in males.

**^+^***p* < 0.05, **^++^***p* < 0.01, **^+++^***p* < 0.001 *versus* same group 3 month old.

by three-way ANOVA with Tukey's post-hoc test.

### 3.6 The basilar artery of young female MFS mice exhibits hypertrophic remodeling

**Fig. 8:**
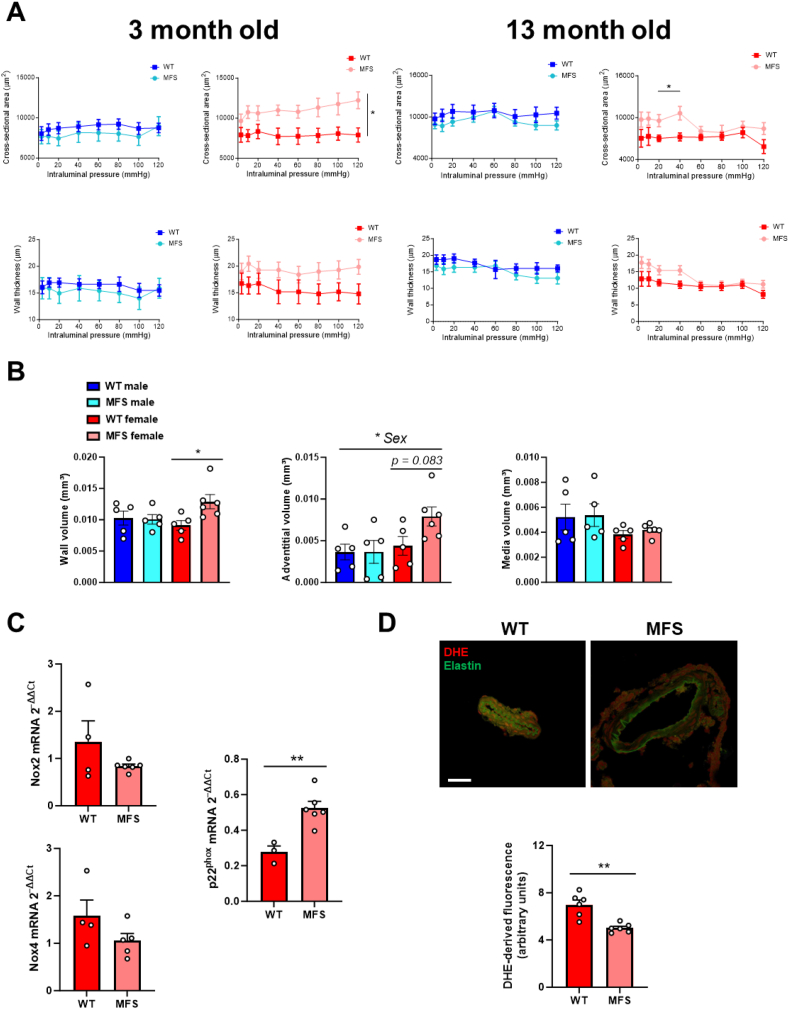
Structural properties, cell nuclei distribution, mRNA expression levels of the NADPH oxidase isoforms Nox2 and Nox4, and NADPH oxidase subunit p22^phox^, and dihydroethidium (DHE)-derived fluorescence in cerebral arteries from 3- and 13-month-old male and female wild-type (WT) and Marfan syndrome (MFS) mice. (A) Basilar artery cross-sectional area (CSA)-intraluminal pressure and wall thickness-intraluminal pressure under passive conditions (0 Ca^2+^-Krebs-Henseleit solution). Results are the mean ± SEM of *n* = 7–8 (WT male), *n* = 5 (MFS male), *n* = 4–6 (WT female), and *n* = 5–8 (MFS female). ∗*p* < 0.05 (3-month-old) by repeated measures two-way ANOVA (genotype factor) or ∗*p* < 0.05 (13-month-old) by repeated measures two-way ANOVA with Bonferroni's post-hoc test. (B) Comparative analysis of cell nuclei distribution by confocal microscopy in basilar arteries of 3-month-old male and female WT and MFS mice. Results are the mean ± SEM with each data point representing an animal. ∗*p* < 0.05 by two-way ANOVA with Bonferroni's post-hoc test. (C) Nox2, Nox4, and p22^phox^ mRNA expression levels in cerebral arteries of 3-month-old female WT and MFS mice. Results are the mean ± SEM with each data point representing an animal. ∗∗*p* < 0.01 by the unpaired Student's t-test. (D) Representative photomicrographs (top) and quantification (bottom) of fluorescence (red) intensity in confocal basilar artery sections from 3-month-old female WT and MFS mice labeled with the oxidative dye DHE. Natural autofluorescence of elastin (green) is also shown. Scale bar, 50 μm. Results are the mean ± SEM with each data point representing an animal. ∗∗*p* < 0.01 by the unpaired Student's t-test.

To explore potential factors contributing to increased MFS mouse neuroinflammation, we evaluated basilar artery properties in WT and MFS mice. Basilar artery diameter was assessed across a pressure range of 3–120 mmHg under fully relaxed conditions (0 Ca^2+^-KH solution) in 3- and 13-month-old WT and MFS mice of both sexes (Fig. 8). The external and lumen diameters in WT mice did not differ from those in MFS mice regardless of age (results not shown). Young female MFS mice showed a significant enlargement of the cross-sectional area over the pressure range compared with WT mice. However, this difference was not observed in MFS males (Fig. 8A). Conversely, in 13-month-old female MFS mice, an enlargement in the cross-sectional area only happened at an intraluminal pressure range of 20–40 mmHg (Fig. 8A).

Assessment of the passive mechanical properties of the basilar artery yielded similar results in 3-month-old mice. However, a divergent response was observed in older (13-month-old) female MFS mice, wherein an increase in wall strain was noted at intraluminal pressures ranging from 80 to 120 mmHg compared with WT mice (Supplementary Fig. 8A). These changes did not occur in male MFS mice. The stress-strain relationship (Supplementary Fig. 8B) and its corresponding β-values were comparable in all mice (results not shown). Collectively, these slight increases in basilar artery elasticity (*i.e.*, increased wall strain) that occurs under physiological to supraphysiological intraluminal pressures in aged female MFS mice are expected to counteract wall hypertrophy.

Examination of cell nuclei distribution in pressurized basilar arteries of 3-month-old mice confirmed heightened basilar artery walls in female MFS mice, characterized by an enlargement of the wall (*p* < 0.05) and adventitial (*p* = 0.083) volume compared with female WT mice (Fig. 8B). As anticipated, no alterations were observed in male MFS mice. However, these changes in nuclei distribution in female MFS mice were not accompanied with a higher number of smooth muscle (WT: 2503 ± 68, *n* = 5; MFS: 3067 ± 284, *n* = 6) or adventitial (WT: 1210 ± 256, *n* = 5; MFS: 2169 ± 520, *n* = 6) cells. Furthermore, basilar artery hypertrophy was associated with increased NADPH oxidase subunit p22^phox^ mRNA expression in cerebral arteries from young MFS females, while Nox2 and Nox4 expression remained unchanged (Fig. 8C). Notably, analysis of DHE-derived fluorescence in basilar arteries as a measure of *in situ* oxidative stress levels showed a significant reduction in oxidative stress in arteries from MFS female mice (Fig. 8D), which was not associated with changes in phospho-NRF2 expression (Supplementary Fig. 8C). In conclusion, our findings show that the basilar artery in female MFS mice has a thicker wall, primarily due to an enlarged adventitial layer, and this remodeling is accompanied by a compensatory reduction in oxidative stress.

### 3.7 Cardiovascular health worsens similarly across all groups following transient BCCAO

To explore whether brain alterations observed in MFS mice could increase susceptibility to worsened post-ischemic damage, we subjected 3-month-old male and female WT and MFS mice to a BCCAO (5 min) followed by three days of reperfusion. The experimental design is shown in Supplementary Fig. 1. After BCCAO, the survival rates were not statistically different across the groups: male WT (36 %), male MFS (58 %), female WT (60 %), and female MFS (27 %). As a measure of well-being, we assessed the body weight and SBP of surviving mice (Table 3). Body weight remained stable after BCCAO. SBP levels significantly decreased in all experimental groups one day post-BCCAO but showed signs of recovery by the third day, with differences no longer statistically significant. Overall, these findings suggest a comparable decline in cardiovascular health across all groups following transient ischemia.

**Table 3:** Physiological parameters one day before (Day −1) and after (Day +1) bilateral common carotid artery occlusion, and before euthanasia (Day +3).

	WT male (*n* = 5)	MFS male (*n* = 7)	WT female (*n* = 6)	MFS female (*n* = 3)
**Body weight (g)**				
Day −1	26.1 ± 1.1	28.3 ± 1.1	20.9 ± 1.0	20.4 ± 1.9**∗∗**
Day +1	23.6 ± 1.0	24.6 ± 1.5	18.9 ± 0.8	19.6 ± 1.4
Day +3	24.3 ± 1.5	25.8 ± 1.4	18.5 ± 1.1**∗**	18.4 ± 0.4**∗**
**Systolic blood pressure (mmHg)**				
Day −1	108.4 ± 5.0	115.1 ± 2.7	108.0 ± 2.1	109.3 ± 1.9
Day +	89.7 ± 3.5**^+^**	94.6 ± 2.4**^+++^**	86.3 ± 3.2**^+++^**	83.0 ± 2.1**^++^**
Day +3	100.8 ± 4.3	99.6 ± 2.1	94.0 ± 5.6	96.7 ± 3.8

Results are the mean ± SEM. ∗*p* < 0.05, ∗∗*p* < 0.01 *versus* the same group in male; ^+^*p* < 0.05, ^++^*p* < 0.01, ^+++^*p* < 0.001 *versus* Day −1 by three-way ANOVA with Tukey's post-hoc test.

### 3.8 MFS mice show poorer behavioral outcomes following transient BCCAO

**Fig. 9:**
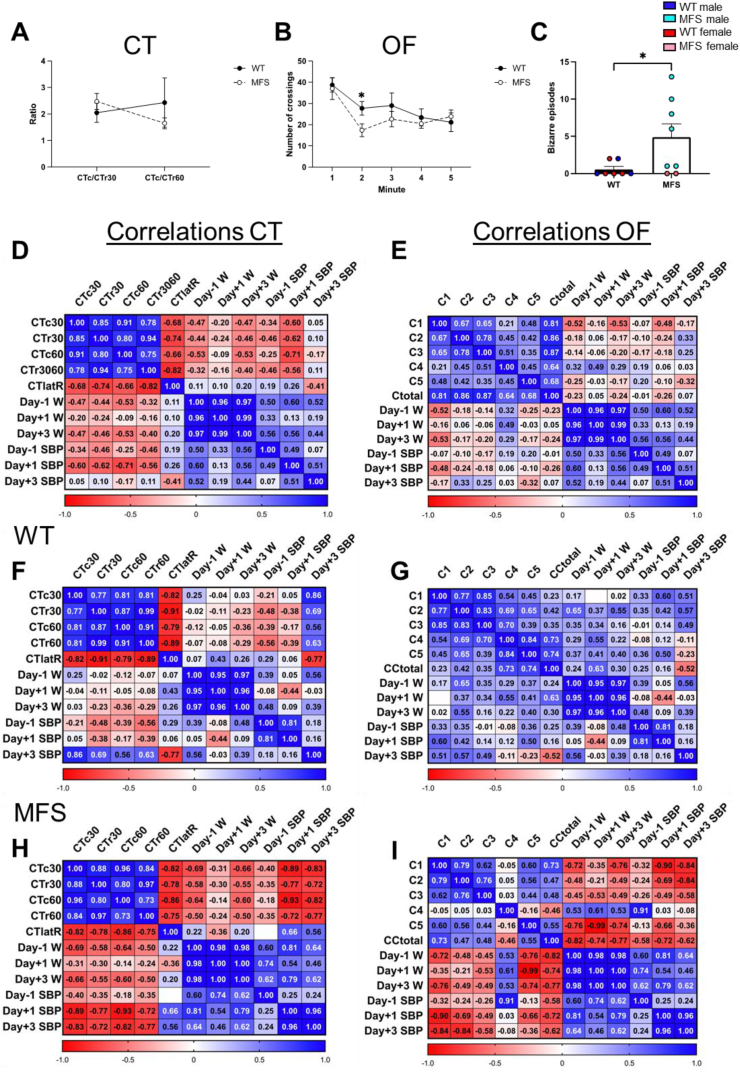
Behavioral assessment and functional correlations between behavior and systolic blood pressure (SBP) and body weight (W) variables in 3-month-old male and female wild-type (WT) and Marfan syndrome (MFS) mice submitted to transient bilateral common carotid artery occlusion. (A) The horizontal and vertical activity ratio in the corner test (CT). (B) Horizontal activity ethogram on the open field (OF) test. (C) Bizarre behavior episodes during OF. Different colors identify male and female WT and MFS groups. Results are the mean ± SEM of 7–8 animals. ∗*p* < 0.05 by the unpaired Student's t-test. (D) Heat map of correlations between behavioral variables and SBP and W variables in the CT. (E) Heat map of correlations between behavioral variables and SBP and W variables in the OF. The color scale shows the correlation intensity between +1 and −1, with positive correlations in blue and negative in red. Additional heat maps of correlations are shown for both behavioral tests separated by genotype, WT (F and G), and MFS (H and I).

Behavioral analysis was conducted to assess whether MFS mice exhibit heightened susceptibility to neurological issues induced by transient BCCAO (Fig. 9). Six of the 21 animals assessed (4/11 WT; 2/10 MFS) exhibited a low behavioral profile suggestive of sickness behavior and, therefore, were excluded from the behavioral data analysis. In the CT, there was a trend toward reduced exploratory activity in MFS mice compared with WT counterparts, although this difference did not reach statistical significance (Fig. 9A). Similarly, the horizontal activity ethogram (OF) revealed decreased crossings in MFS mice compared with WT mice 2 min after test onset (Fig. 9B), indicative of reduced exploratory behavior. Notably, MFS mice exhibited an increased frequency of bizarre behavior episodes (Fig. 9C), suggesting anxiety-like behaviors. To further comprehend the impact of behavioral changes, correlations with other well-being variables, such as body weight and SBP, were calculated. Initially, the entire population was assessed in both behavioral tests (Fig. 9D and E), followed by separate analyses for each genotype (Fig. 9F–I). Heat maps revealed that most behavioral variables were interconnected, suggesting homogeneity in behavior that aligns with expectations. These behavioral variables were also correlated with body weight and SBP. Furthermore, body weight and SBP variables were also correlated one to each other, indicating an interdependence between behavior, body weight, and SBP. Upon considering genotypes separately, differences in the correlations of behavioral variables with body weight and SBP were evident. Specifically, the MFS genotype exhibited stronger negative correlations in the case of the CT and a shift from positive (in WT animals) to negative (in MFS animals) in the OF. Additionally, we examined potential sex-based differences by separately evaluating correlations for males and females. However, no significant differences were observed between the two groups, possibly because the low number of females is not sufficient to obtain statistically strong correlations. Taken together, these findings suggest that after BCCAO, MFS mice show an altered behavioral phenotype, as evidenced by increased bizarre behaviors, which potentially contribute to a decreased exploratory activity.

### 3.9 Brain and basilar artery oxidative stress is similar across all groups after transient BCCAO

We assessed superoxide anion production in the brain after BCCAO by measuring 2-EOH levels (Supplementary Fig. 9A) and no significant changes were observed. Likewise, lipid peroxidation (Supplementary Fig. 9B) and NO levels (Supplementary Fig. 9C) showed no significant differences between experimental groups. In addition, DHE-derived fluorescence in the basilar arteries indicated similar oxidative stress levels (Supplementary Fig. 9D). Likewise, phospho-NRF2 immunofluorescence analysis in the hippocampus following BCCAO showed no significant differences between MFS and WT mice, regardless of sex or age (Supplementary Fig. 10).

### 3.10 MFS mice display increased neurodegeneration and heightened neuroinflammation after transient BCCAO

**Fig. 10:**
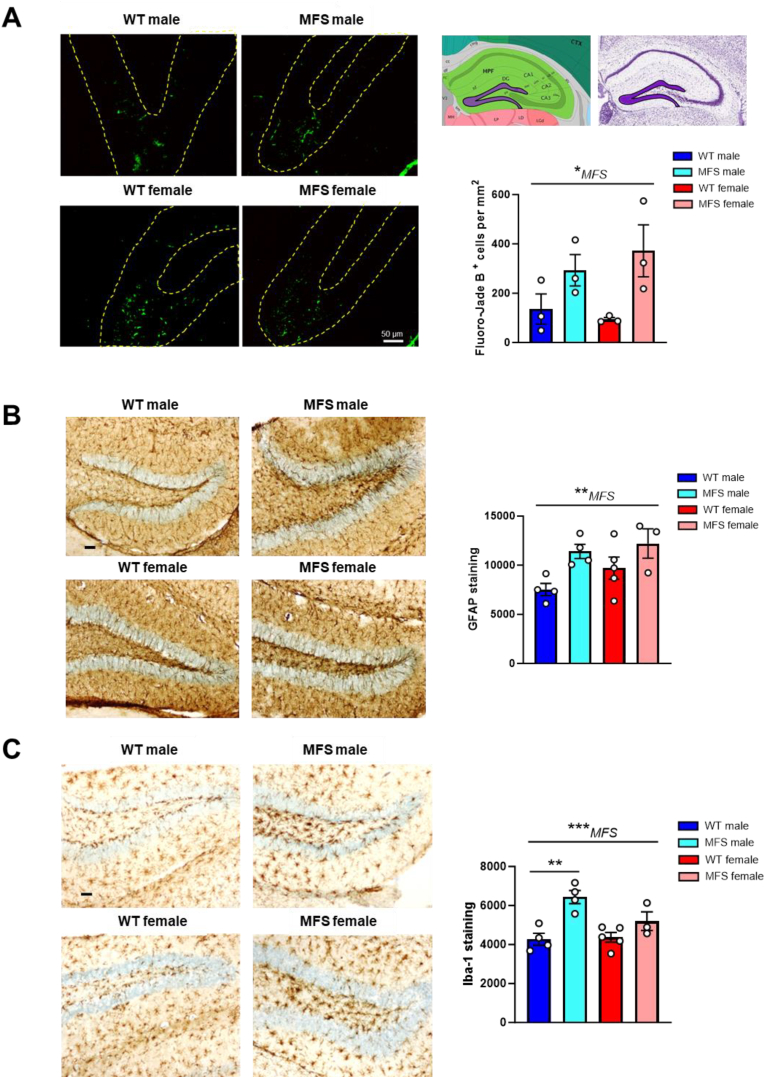
Hippocampal neuronal degeneration and neuroinflammatory response in 3-month-old male and female wild-type (WT) and Marfan syndrome (MFS) mice subjected to transient bilateral common carotid artery occlusion. (A) On the right are representative coronal sections of the mouse brain at the level of the hippocampus (adapted from the Allen Mouse Brain Atlas https://mouse.brain-map.org/static/atlas). The violet-shaded areas indicate the granular layer of the dentate gyrus, where Fluoro-Jade B fluorescent staining was performed. On the left are representative images of Fluoro-Jade B-positive degenerating neurons (green fluorescence). Yellow dashed lines delineate the granular layer of the dentate gyrus. Bar graphs show the density results of Fluoro-Jade B-positive neurons in the dentate gyrus. (B) Representative images of astrocytic GFAP immunostaining in the dentate gyrus. Bar graphs show the results of GFAP immunostaining obtained by densitometry. (C) Representative images of microglial Iba-1 immunostaining in the dentate gyrus. Bar graphs show the results of Iba-1 immunostaining obtained by densitometry. Results are the mean ± SEM. Data points represent the number of animals. ∗*p* < 0.05, ∗∗*p* < 0.01, ∗∗∗*p* < 0.001 by two-way ANOVA with Tukey's post-hoc test. Scale bar, 50 μm.

Transient BCCAO leads to neuronal cell death, particularly in vulnerable areas as the hippocampus [23]. To examine hippocampal neurodegeneration following BCCAO, we utilized Fluoro-Jade B fluorescent staining (Fig. 10A). Given that most Fluoro-Jade B-positive cells were found in the dentate gyrus, quantitative analysis was focused only in this region. Our findings reveal that transient BCCAO induces greater hippocampal neurodegeneration in both male and female MFS mice compared with WT counterparts (Fig. 10A). In addition, we investigated the neuroinflammatory response post-BCCAO by assessing GFAP (Fig. 10B) and Iba-1 (Fig. 10C) immunostaining in the hippocampus. Following BCCAO, increased GFAP and Iba-1 expression were observed in MFS mice compared with WT mice, indicating an enhanced neuroinflammatory response in post-BCCAO MFS animals. Taken together, these findings indicate that MFS mice exhibit heightened susceptibility to ischemic brain injury after BCCAO.

## 4 Discussion

The most critical clinical concern in MFS is aortic dissection, a life-threatening condition. In addition, various other clinical features linked to MFS impact significantly on the lives of patients. Notably, individuals with MFS often exhibit distinctive psychiatric and neuropsychological alterations, which profoundly influence or even determine their quality of life and overall well-being [[18], [19], [20], [21]]. Critically, before the present study, there was a notable absence of preclinical evidence concerning brain tissue implications in this syndrome. Here, we present the first comprehensive evidence of brain alterations in a mouse model of MFS (*Fbn1*^*C1041G/+*^) [22] demonstrating sex- and age-specific changes in TGF-β1 and ECM turnover pathways, while neuroinflammation is consistently observed across all MFS mice, regardless of sex and age. Despite increased NADPH oxidase activity, overall brain redox stress levels remain largely unchanged, likely due to compensatory antioxidant responses, including spermine in males and estrogens in females. Nonetheless, the corpus callosum in male MFS mice still shows evidence of increased redox stress damage. Besides, basilar artery hypertrophic remodeling is observed only in young female mice. Importantly, following BCCAO, both young male and female MFS mice show increased neuroinflammation and worsened post-ischemic brain injury. Together, these findings highlight the underlying brain pathology in MFS, which appears early in life and persists into later stages. This suggests that MFS patients, alongside the well-established aortic complications, have an intrinsic predisposition to post-ischemic brain damage.

### 4.1 TGF-β signaling and ECM turnover in MFS mouse brains: preservation in youth and disruption in adulthood

Due to *Fbn1*-pathological variants, latent TGF-β binding proteins fail to retain this cytokine in the ECM (fibrillin-1 microfibrils), leading to persistently abnormal TGF-β hypersignaling and aortic aneurysm formation [49]. TGF-β signals through SMAD proteins (canonical pathway), which, when phosphorylated, translocate to the nucleus to promote gene transcription [15]. In addition, TGF-β also activates noncanonical signaling cascades involving the neurogenic locus notch homolog (NOTCH) protein, mitogen-activated protein kinase/extracellular signal-regulated kinase (MAPK/ERK), RAC serine/threonine protein kinase (AKT), nuclear factor kappa B, and WNT [15]. Moreover, in MFS aortic disease, the role (protective or detrimental) of TGF-β signaling is time-dependent [50,51]. On the other hand, TGF-β signaling in the brain is essential for neurodevelopment and responding to brain injury [52]. There is contrasted evidence that both the magnitude and timing of TGF-β signaling play crucial roles in determining whether this cytokine exacerbates neuroinflammation or aids in its resolution/attenuation and/or prevention [52]. For instance, mice with homozygous astrocyte-targeted overproduction of TGF-β exhibit meningeal and membrane blood vessel thickening, ventricle enlargement, and hydrocephalus, whereas these effects were not observed in mice with heterozygous transgene expression [16,17].

In our study, we observed a consistent decrease in the expression of some TGF-β pathway genes in young (3-month-old) male MFS mice, with similar but less pronounced effects in aged (13-month-old) female MFS mice. Strikingly, these changes did not correlate with brain alterations in phosphorylated SMAD2 and ERK1/2 protein expression, which are key mediators of TGF-β signaling. The apparent disconnection between unaltered TGF-β1 levels and the downregulation of TGF-β pathway genes in young male MFS mice suggests a compensatory negative feedback mechanism, where earlier (*i.e.*, before 3 months of age) heightened TGF-β1 signaling leads to transcriptional repression of pathway components, serving as a protective mechanism to prevent excessive activation of TGF-β signaling. Interestingly, in aged MFS mice, we identified a decrease in TGF-β1 expression in the hippocampus compared to WT mice regardless of sex. This decrease in TGF-β1 expression could reflect a compensatory downregulation of the cytokine to counterbalance chronic inflammation [53]. A slight downregulation of the TGF-β signaling pathway transcriptome was observed in aged female MFS mice, which concomitantly occurred with reductions in both brain TGF-β1 levels and circulating estradiol levels. Previous studies have suggested that estrogens inhibit TGF-β signaling [54]. The observed decrease in estrogen levels in aged female MFS mice could contribute to the compensatory increase in TGF-β signaling at later stages (*i.e.*, after 13 months of age).

In MFS, TGF-β hypersignaling disrupts the balance of gene expression involved in maintaining the structural ECM integrity, such as collagens, elastin, fibulins, and integrins, and tightly related pathways such as MMPs and tissue inhibitors of MMPs [15]. Accordingly, transcriptome analysis of ECM turnover pathways showed a significant enhancement in expression in both young male and aged female MFS mice. The observed sex-by-genotype interaction in collagen I and pro-MMP9 levels suggests that mutated fibrillin-1 contribute to some extent to the sex-specific vulnerability of the ECM homeostasis independent of age. Notably, the increased pro-MMP9 expression and slightly elevated collagen I levels in aged female MFS mice, coupled with the activation of ECM turnover pathways, point to a potential shift toward fibrotic-like ECM remodeling in the brain. We suggest that the decline in estrogen levels in aged female MFS mice could contribute to the establishment of these alterations. This dysregulation could have significant implications for overall brain homeostasis in MFS, particularly in aged females, who may be more susceptible to pathological ECM alterations that compromise tissue integrity and repair mechanisms, ultimately increasing the likelihood of brain damage.

### 4.2 Adaptive redox responses in MFS mice with region-specific redox stress elevations in males

Maintaining redox balance in the brain is vital for cellular function, neuronal integrity, and protection against oxidative damage, ultimately supporting brain health and cognitive performance. Previous studies have demonstrated elevated redox stress in MFS, particularly in aorta [44,45] and cerebral arteries [13]. In the present study, we observed increased Nox4 expression in the brains of both male and female MFS mice, suggesting upregulation of the Nox4 pathway in the brain, consistent with findings in the aorta [45] and cerebral arteries [13]. Despite these transcriptional changes and increased NADPH oxidase activity, we did not observe widespread oxidative or nitrosative stress, as superoxide anion levels, lipid peroxidation, and NO levels remained unchanged. Interestingly, we observed region-specific alterations in redox stress damage in male MFS mice, with elevated lipid oxidation and increased β/α protein folding in the corpus callosum. In contrast, female MFS mice showed no significant redox stress damage. These findings suggest that while the MFS brain does not exhibit widespread redox stress, certain regions, particularly in males, could be more susceptible to redox stress-associated damage. Notably, the elevated spermine levels in MFS males point to an adaptive mechanism aimed at counteracting redox stress. Spermine is a noteworthy antioxidant as it activates the NRF2 pathway [55,56], scavenges reactive oxygen species [57,58], and enhances the activity of various endogenous antioxidant enzymes [59]. In fact, the lack of alterations in phospho-NRF2 in the MFS brain, contrasting with findings in the aorta [46], further supports the presence of a healthy antioxidant defense system. Besides, the lack of significant changes in brain redox stress in female MFS mice could be attributed to the protective role of estrogens, with sustained estradiol levels in young female MFS mice potentially contributing to a reduced vulnerability to redox damage.

These findings underscore the complex, sex-specific regulation of brain redox stress in MFS, where compensatory antioxidant responses seem to mitigate early-stage damage. However, the regional susceptibility to redox stress damage in male MFS mice suggests that, despite compensatory mechanisms, certain regions remain more vulnerable. Understanding these region-specific alterations could offer valuable insights into targeted therapeutic strategies for mitigating potential localized redox stress in the MFS brain.

### 4.3 Both male and female MFS mice show increased neuroinflammation

Prior experimental evidence has linked MFS with increased inflammation [60,61]. However, no studies have been carried out to thoroughly characterize neuroinflammation either in MFS patients or preclinical models. The controlled inflammatory response is crucial for maintaining brain function, however, excessive neuroinflammation can lead to neuronal damage and increased susceptibility to brain injury. In fact, redox stress and neuroinflammation are interconnected, creating a vicious cycle that exacerbates neuronal damage [62]. In our study, transcriptomic analysis revealed a consistently increased expression of inflammatory pathways in MFS mice, regardless of sex and age. In addition, we observed the appearance of gliosis in both male and female MFS mice, a process characterized by augmented astrocyte and microglia activation/reactivity. Both collagen I and pro-MMP9 are regulated by TGF-β signaling, a key pathway in ECM remodeling and fibrosis. The sex per genotype-dependent changes in collagen I expression observed in MFS mice have significant implications for neuroinflammation. The trend toward an increased fibrotic response in aged female MFS mice was associated with heightened expression of GFAP and Iba-1, suggesting an exacerbation of neuroinflammatory processes. Conversely, the attenuated fibrotic response in male MFS mice could indicate an adaptive mechanism to limit neuroinflammation, which ultimately proved ineffective. The observed sex-dependent differences in ECM dynamics could therefore stem from differential transcriptional regulation of the TGF-β pathway, potentially influenced by the decline in estrogen levels [54]. These findings underscore the importance of sex as a determinant of the mechanisms driving neuroinflammation in MFS and suggest that aged female MFS mice could be more susceptible than males to pathological ECM alterations that contribute to this process.

On the other hand, we suggest that neuroinflammation in young male MFS mice could be partly enhanced by brain region-specific redox stress and the A1 astrocyte response, which is known to be activated in reaction to neuroinflammatory stimuli [63]. Of note, the present study suggests that supplementing with exogenous spermine could be a potential therapeutic strategy to reduce excessive inflammation in male MFS mice, particularly considering the previously documented anti-inflammatory properties of spermine [64]. However, unlike male mice, increased neuroinflammation in female MFS animals was not associated with major alterations in redox stress. Another factor exacerbating brain inflammation is the infiltration of peripheral immune cells, cytokines, and other inflammatory mediators into the parenchyma [65]. This could result from BBB dysfunction, as previously reported in female *ApoE*^*−/−*^*Fbn1*^*C1041G/+*^ mice fed a Western-type diet [12]. While here we did not specifically investigate BBB disruption, our study provides indirect evidence suggesting its dysfunctional implications [66]. The neuroinflammatory cascade associated with BBB injury is strongly correlated with, among other factors, increased levels of IL-1β [67]. For example, we noted elevated mRNA levels of IL-1β (immune response and inflammation) and CD68 (microglial activation and macrophage recruitment) in the brains of aged female mice, regardless of genotype. This suggests that MFS in females does not modify the naturally occurring changes in these inflammatory markers with aging, which is relevant for maintaining their susceptibility to brain damage.

In addition, our results show that young female MFS mice exhibit basilar artery hypertrophic remodeling. Wall thickening during arterial remodeling reduces tensile stress but may induce turbulent flow, locally altering shear stress patterns and leading to blood flow impairment [68]. These results suggest potential sex-dependent disruptions in cerebral blood flow as well. This, in turn, contributes to inflammatory processes in the brain and BBB dysfunction [69]. Notably, we observed increased expression of the cerebral artery NADPH oxidase subunit p22^phox^ in young female MFS mice. However, instead of increasing oxidative stress, these changes suggest a compensatory endogenous antioxidant response, as *in situ* oxidative stress in the basilar artery was reduced. Furthermore, the absence of alterations in basilar artery phospho-NRF2 expression points to a healthy antioxidant response, reinforcing the idea that, like observed in the brain, vascular redox balance alterations are effectively mitigated by local antioxidant defense mechanisms. The vascular remodeling observed in young female MFS mice and the compensatory reduction in oxidative stress would represent an adaptive response to mitigate further damage. It is noteworthy that the simultaneous observation of decreased circulating estradiol levels in aged female MFS mice and the absence of basilar artery remodeling at this age suggest that estrogens play a role in determining basilar artery remodeling. This hypothesis is further supported by previous reports highlighting the crucial role of estrogens in flow-mediated remodeling of mesenteric resistance arteries in rodents exposed to high flow [70].

Overall, this study underscores the presence of elevated neuroinflammation in MFS mice, irrespective of sex and age, while also highlighting potential differences in its underlying mechanisms between males and females. In males, region-specific increases in brain redox stress and the activation of neuroinflammatory pathways may drive neuroinflammation. In females, however, neuroinflammation appears to be more closely linked to pathological ECM alterations, possibly disrupted cerebral artery blood flow, and the infiltration of peripheral immune cells and inflammatory mediators due to BBB dysfunction. Nevertheless, further research is required to better understand these associations.

### 4.4 Increased post-ischemic brain injury in MFS mice

Brain ischemia, a significant contributor to mortality and disability worldwide, encompasses various conditions where blood flow to the brain is either reduced or halted. One form, global brain ischemia, occurs due to factors like cardiac arrest, shock, carotid occlusion, hypotension, asphyxia, or anemia. BCCAO is a widely used method to induce global cerebral ischemia in animal models, leading to delayed neuronal death and neurological impairments, particularly in brain regions like the hippocampus and retina [23]. The resulting brain damage involves redox stress, inflammation, and BBB dysfunction, definitively contributing to neuronal damage and white matter lesions. To assess whether baseline brain abnormalities in MFS mice increase the risk of cerebral injuries following an ischemic episode, we subjected the mice to BCCAO. Firstly, to understand the potential impact of ischemic injury on the psychological, social, and behavioral aspects of the syndrome, we characterized mouse behavior using a battery of well-defined tests. After transient BCCAO, MFS mice exhibited more frequent or severe episodes compared to WT mice, which affected their normal exploratory behavior. Some extreme cases of MFS have been linked to schizophrenia and other psychiatric disorders [71,72]. All of these are consistent with the bizarre behavior exhibited by MFS mice, possibly indicating illness-related psychosis [73,74]. As the cardiovascular system is one of the principal systems affected in MFS, the relationship between the cardiovascular system status and behavior was also assessed. Cardiovascular and systemic vascular functioning have been repeatedly linked with cognitive performance and behavior, especially in cardiovascular diseases [75,76]. Previous work by the group with a neurodegenerative disease mouse model found that neophobic and anxious-like profiles also involved vascular compensation systems [77]. In the current study, correlation analysis of the variables did indeed suggest a strong relationship between behavioral variables and blood pressure in ischemic MFS animals, which were not present in ischemic WT animals. They showed a relation with neophobia (freezing), anxiety, stress behaviors (high vertical activity in the OF), and bizarre behavior. These results indicate that, after an ischemic insult, the MFS genotype affects both neurological aspects and the cardiovascular system. Further research will be needed to unveil the underlying mechanism(s) leading to this relationship and to investigate in detail the sex factor, which could not be performed here due to the low number of animals studied.

Next, we investigated whether BCCAO-induced neurological alterations in MFS mice were associated with increased brain damage. Global redox stress levels in the brain and basilar arteries remained similar between groups following BCCAO. However, these findings do not rule out the possibility of brain region-specific redox stress alterations or basilar artery remodeling after BCCAO, which were not explored in this study. We then focused on the hippocampus, where baseline neuroinflammation was particularly prominent, to assess the extent of cerebral ischemic damage. Our results show that ischemic injury was more severe in the hippocampus of MFS mice, leading to increased neuronal degeneration and heightened activation of astrocytes and microglia. The present study suggests that the increased susceptibility to ischemic damage in the brains of MFS mice (both males and females) could be partly attributed to preexisting neuroinflammation in the hippocampus and other brain areas.

### 4.5 Limitations of the study

This study has several limitations worth noting. First, the mechanisms underlying TGF-β signaling alterations were not fully characterized. Although we observed transcriptional changes indicative of regulatory imbalances, a reduction in brain TGF-β1 levels, and alterations in several direct targets of TGF-β signaling, further research is needed to clarify the temporal dynamics and functional impact of TGF-β pathway modulation in the brain across age and sex in MFS, including the potential influence of estrogen. Second, the role of NADPH oxidase in redox regulation of the MFS brain remains incompletely understood. Although isoform-specific expression changes, increased activity, and compensatory antioxidant responses were observed, the mechanisms and significance of NADPH oxidase modulation in the brain require further investigation, both under baseline conditions and in the presence of comorbidities or physiological stress superimposed on MFS. Lastly, our study focused on the acute (1 day) and subacute (3 days) phases following transient cerebral ischemia. Due to the high mortality associated with the model at later stages, long-term recovery could not be assessed. Future studies using survival-optimized models of brain injury are necessary to evaluate the extended trajectory of inflammatory and neurovascular responses in MFS animals.

### 4.6 Conclusion

In summary, these findings indicate that both male and female MFS mice exhibit heightened susceptibility to ischemic damage, even with the activation of antioxidant responses, such as increased spermine levels in males and the protective presence of estrogens in young females. This increased susceptibility is likely due, in part, to an underlying basal neuroinflammatory process present from early ages and persisting into later stages. These findings underscore the secondary health risks associated with MFS and emphasize the importance of addressing brain abnormalities to monitor neurological vulnerabilities. Furthermore, the study suggests that targeted interventions aimed at alleviating neuroinflammatory processes are crucial for preserving brain health in these patients.

## CRediT authorship contribution statement

**Gemma Manich:** Writing – review & editing, Visualization, Supervision, Methodology, Investigation, Formal analysis, Data curation. **Belén Pérez:** Writing – review & editing, Visualization, Methodology, Investigation, Formal analysis, Data curation. **Clara Penas:** Writing – review & editing, Visualization, Methodology, Investigation, Formal analysis, Data curation. **Ana Paula Dantas:** Writing – review & editing, Visualization, Methodology, Investigation, Formal analysis, Data curation. **Joana Coutinho:** Writing – review & editing, Methodology, Investigation, Formal analysis. **Paula Sánchez-Bernadó:** Writing – review & editing, Methodology, Investigation, Formal analysis. **Julián García-Aranda:** Writing – review & editing, Methodology, Investigation, Formal analysis. **Juan Fraile-Ramos:** Writing – review & editing, Methodology, Formal analysis. **Núria Benseny-Cases:** Writing – review & editing, Methodology, Formal analysis. **Beatriz Martín-Mur:** Writing – review & editing, Methodology, Formal analysis. **Anna Esteve-Codina:** Writing – review & editing, Methodology, Formal analysis. **Isaac Rodríguez-Rovira:** Writing – review & editing, Methodology, Formal analysis. **Lydia Giménez-Llort:** Writing – review & editing, Visualization, Methodology, Investigation, Formal analysis, Data curation. **Gustavo Egea:** Writing – review & editing, Supervision, Resources, Funding acquisition. **Francesc Jiménez-Altayó:** Writing – review & editing, Writing – original draft, Visualization, Validation, Supervision, Software, Resources, Project administration, Methodology, Investigation, Funding acquisition, Formal analysis, Data curation, Conceptualization.

## Funding

This work was supported by the Ministerio de Ciencia e Innovación and Agencia Estatal de Investigación of Spain [PID2020-113634RB-C22/AEI/10.13039/501100011033 to F.J.-A. and PID2020-113634RB-C21/AEI/10.13039/501100011033 and PID2023-146296OB-I00 to G.E.]; and the 10.13039/501100002809Generalitat de Catalunya [2021 SGR 00969 to F.J.-A. and 2021 SGR 00029 to G.E.].

## Declaration of competing interest

The authors declare that the research was conducted in the absence of any commercial or financial relationships that could be interpreted as a potential conflict of interest.

## Data Availability

Data will be made available on request.
