# Supplementary material for: Sex- and age-dependent neurovascular abnormalities linked to neuroinflammation lead to exacerbated post-ischemic brain injury in Marfan syndrome mice
Source: Redox Biol. 2025 May 7;83:103662. doi: 10.1016/j.redox.2025.103662 (PMC12139021; doi:10.1016/j.redox.2025.103662)
Supplement: Multimedia component 1 [file mmc1.docx]

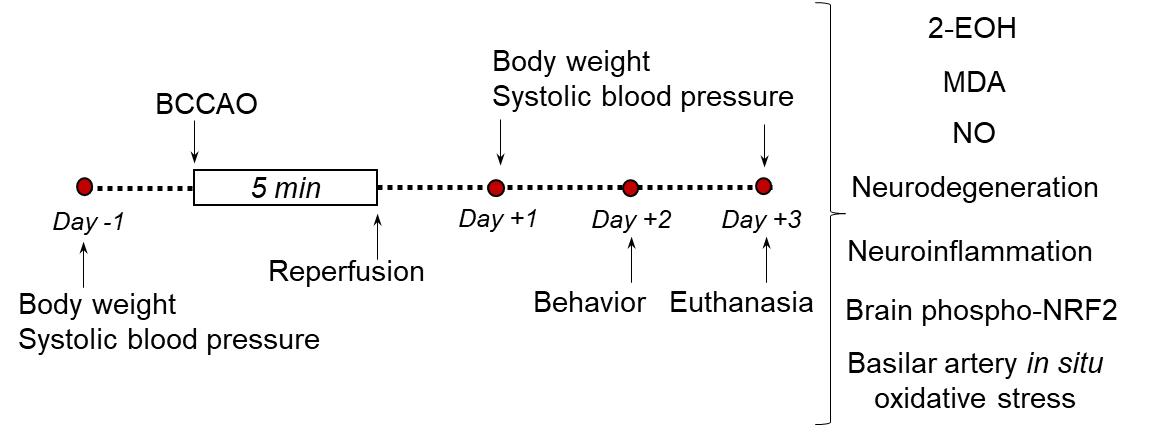


**Supplementary Figure 1.** A schematic diagram illustrating the experimental design of the study investigating the effects of transient global cerebral ischemia induced by bilateral common carotid artery occlusion (BCCAO). Body weight and systolic blood pressure were measured one day before BCCAO, one day after, and before euthanasia on day 3. The animals underwent a 5-min occlusion followed by 3 days of reperfusion. Behavioral tests were performed on day 2 post-BCCAO. On day 3, after euthanasia, various analyses were conducted, including the assessment of 2-hydroxyethidium (2-EOH), malondialdehyde (MDA), and nitric oxide (NO) levels. Neurodegeneration was evaluated using Fluoro-Jade B staining, while brain inflammation was assessed through glial fibrillary acidic protein (GFAP) and ionized calcium-binding adapter molecule 1 (Iba-1) immunohistochemistry. Furthermore, phospho-NRF2 expression was examined via immunofluorescence, and *in situ* oxidative stress in the basilar artery was analyzed using dihydroethidium staining.


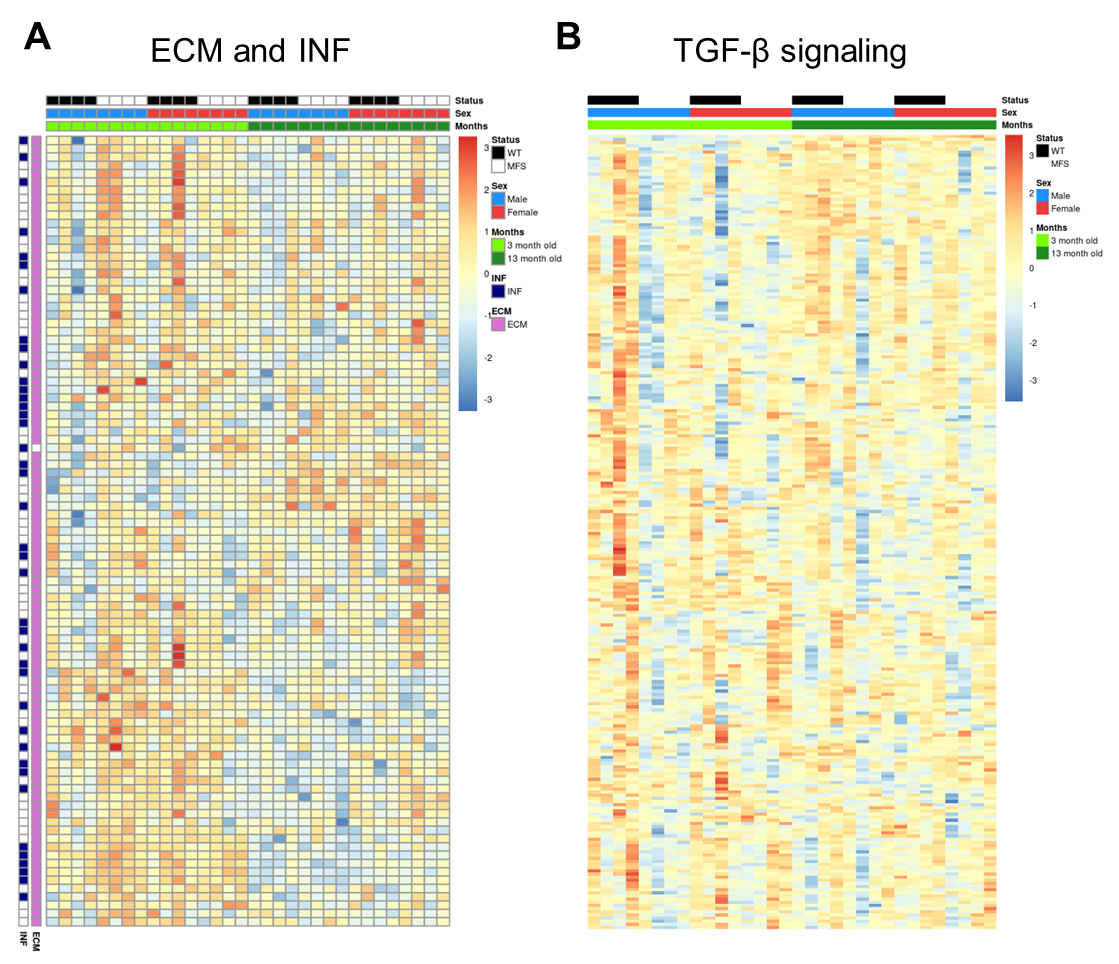
**Supplementary Figure 2.** Heat map depicting the log-normalized expression levels of genes involved in extracellular matrix turnover (ECM) and inflammation (INF) (A) and TGF-β signaling (B) Reactome pathways in the brains of 3- and 13-month-old male and female wild-type (WT) and Marfan syndrome (MFS) mice. Lower and higher expression levels are represented in blue and red, respectively. Each row corresponds to a single gene, and each column represents a sample. *N* = 4 per experimental group.

**
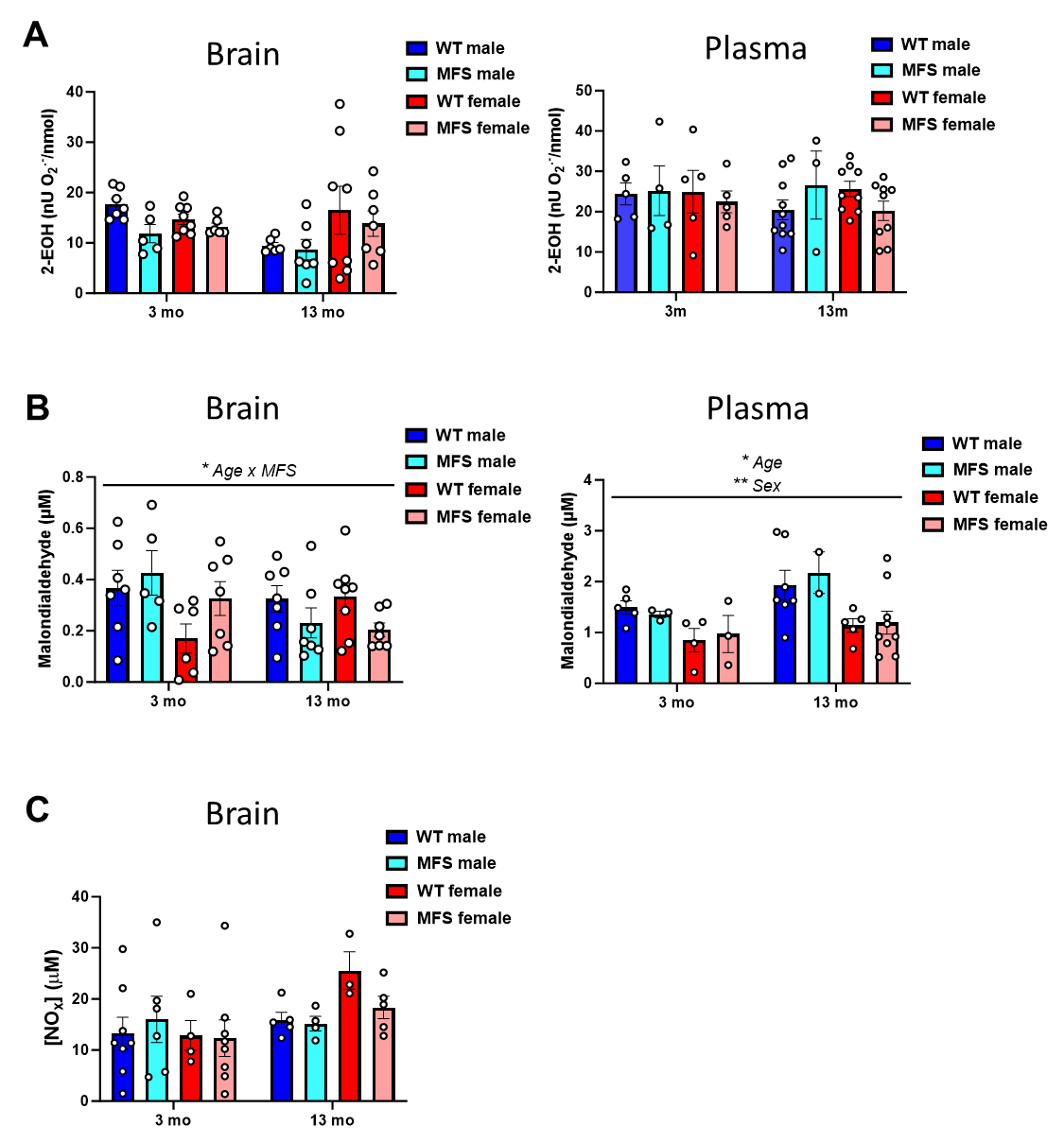
**

**Supplementary Figure 3.** Levels of some representative redox markers measured in brains from 3- and 13-month-old male and female wild-type (WT) and Marfan syndrome (MFS) mice. (A) 2-hydroxyethidium (2-EOH) concentrations analyzed by HPLC, as an indirect indicator of superoxide anion. (B) Malondialdehyde concentrations, as an indirect indicator of lipid oxidation, measured by spectrophotometry. (C) Nitric oxide metabolites (nitrites and nitrates) concentrations measured by spectrophotometry. Results are the mean ± SEM. Each data point represents an animal. **p* < 0.05, ***p* < 0.01 by three-way ANOVA with Tukey's post-hoc test.


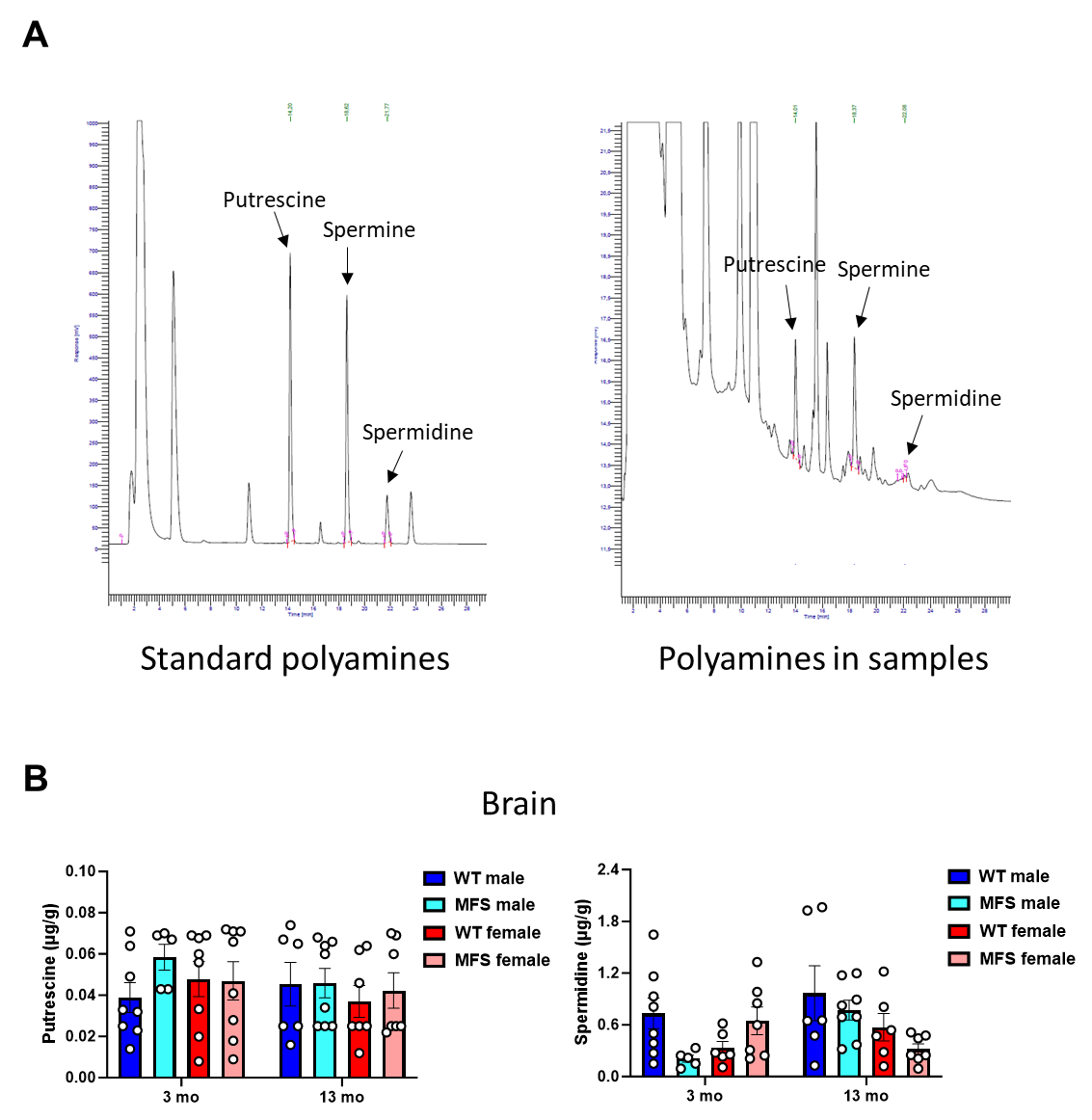


**Supplementary Figure 4.** Detection of polyamines levels in the brain by HPLC. (A) Representative chromatographic traces showing HPLC peaks for putrescine, spermine, and spermidine in a standard sample (left) and a biological sample (right), used for quantification. The retention times for putrescine, spermine, and spermidine were circa 14.0 min, 18.5 min, and 22.0 min, respectively. (B) Putrescine and spermidine concentrations in brains from 3- and 13-month-old male and female wild-type (WT) and Marfan syndrome (MFS) mice. Results are the mean ± SEM. Each data point represents an animal.


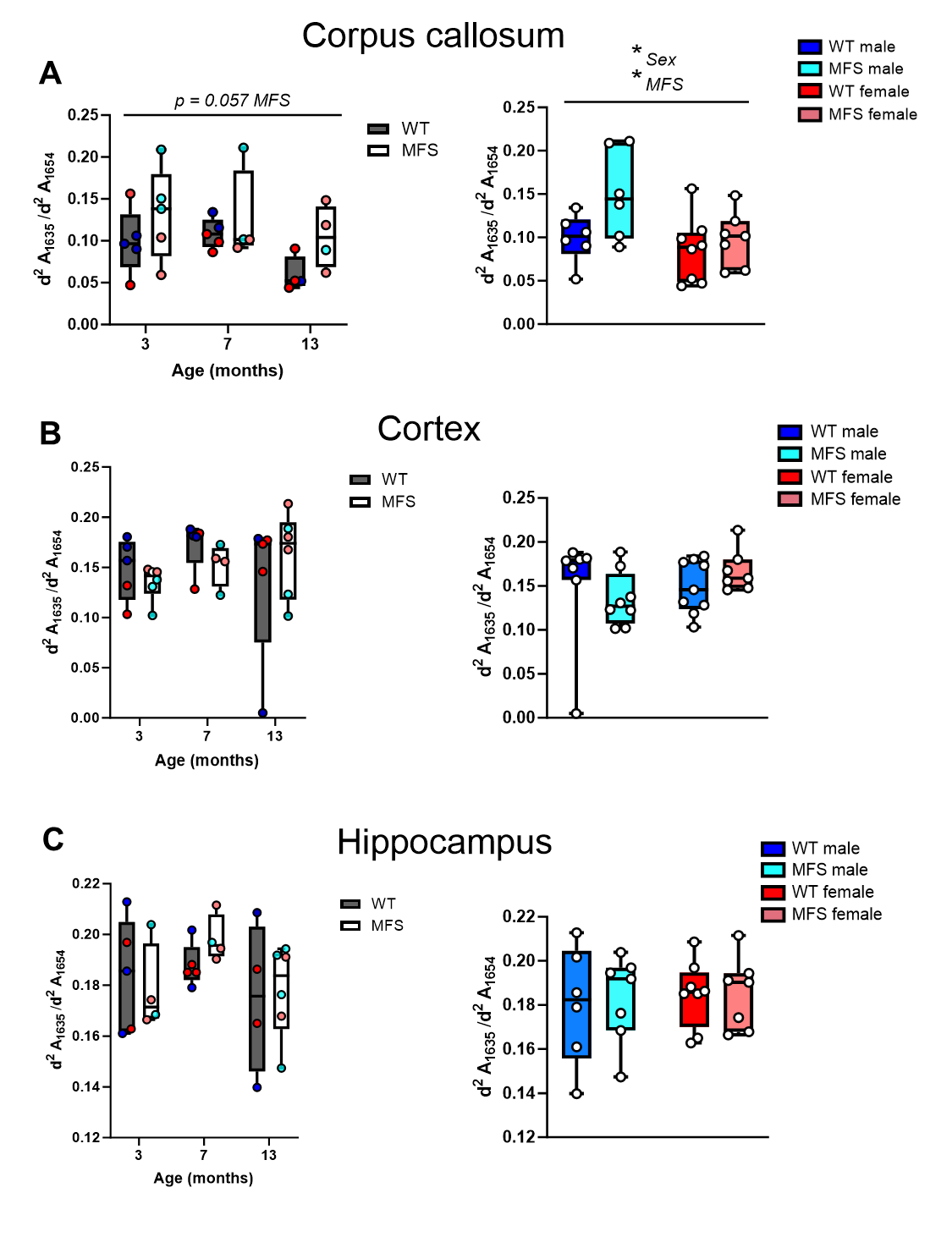


**Supplementary Figure 5.** Representation of the ratio of the second derivative (d^2^A) of the spectra of β-sheet and α-helix (d^2^A1635/ d^2^A1654) as a measure of protein aggregation using μSR-FTIR in (A) corpus callosum, (B) cortex, and (C) hippocampus from 3-, 7-, and 13-month-old male and female wild-type (WT) and Marfan syndrome (MFS) mice. Different colors identify male and female WT and MFS groups. On the right, data from all ages are combined and separated by sex and genotype (MFS). Results are median [Q1; Q3] with each data point representing an animal. **p* < 0.05 by two-way ANOVA with Tukey's post-hoc test.


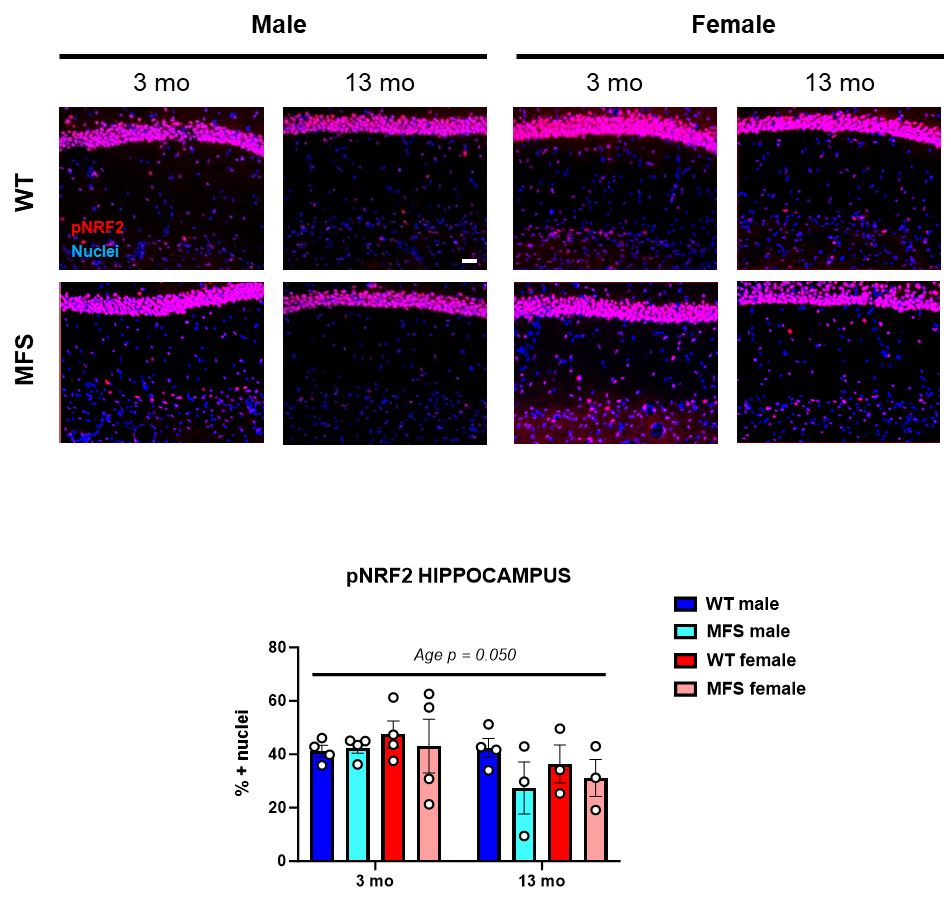


**Supplementary Figure 6.** Expression of phospho-NRF2 (pNRF2) in the hippocampus of 3- and 13-month-old male and female wild-type (WT) and Marfan syndrome (MFS) mice. Representative images of pNRF2 immunofluorescence (red) and nuclei staining (blue; DAPI) in brain hippocampus. Scale bar, 50 µm. Bar graphs show the results of the percentage of pNRF2-positive nuclei in this area. Results are the mean ± SEM with each data point representing an animal. Statistical analysis was performed with three-way ANOVA with Tukey's post-hoc test.


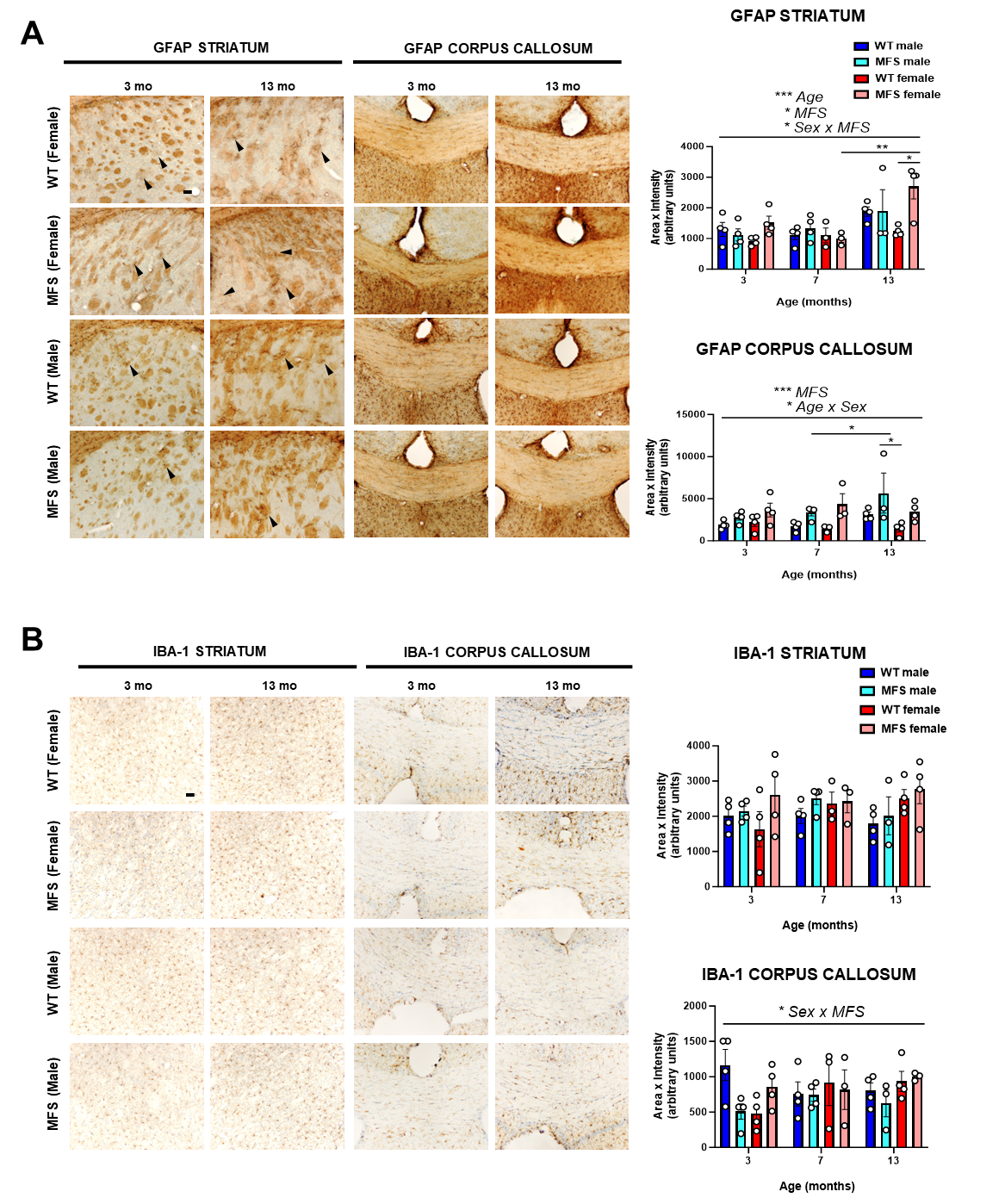


**Supplementary Figure 7.** Glial reactivity in 3-, 7-, and 13-month-old female and male wild-type (WT) and Marfan syndrome (MFS) mice. (A) Representative images of astrocytic GFAP immunostainings of 3 and 13-month-old female and male WT and MFS mice in brain striatum and corpus callosum. Bar graphs show the results of GFAP immunostaining obtained by densitometry in 3-, 7-, and 13-month-old female and male WT and MFS mice. Representative GFAP-positive astrocytes are indicated with arrowheads in the striatum. Scale bar, 50 µm. (B) Representative images of microglial Iba-1 immunostaining of 3 and 13-month-old female and male WT and MFS mice in brain striatum and corpus callosum. Bar graphs show the results of Iba-1 immunostaining obtained by densitometry in 3-, 7-, and 13-month-old female and male WT and MFS mice. Scale bar, 50 µm. Results are the mean ± SEM. Data points represent the number of animals. **p* < 0.05, ****p* < 0.001 by three-way ANOVA with Tukey's post-hoc test.


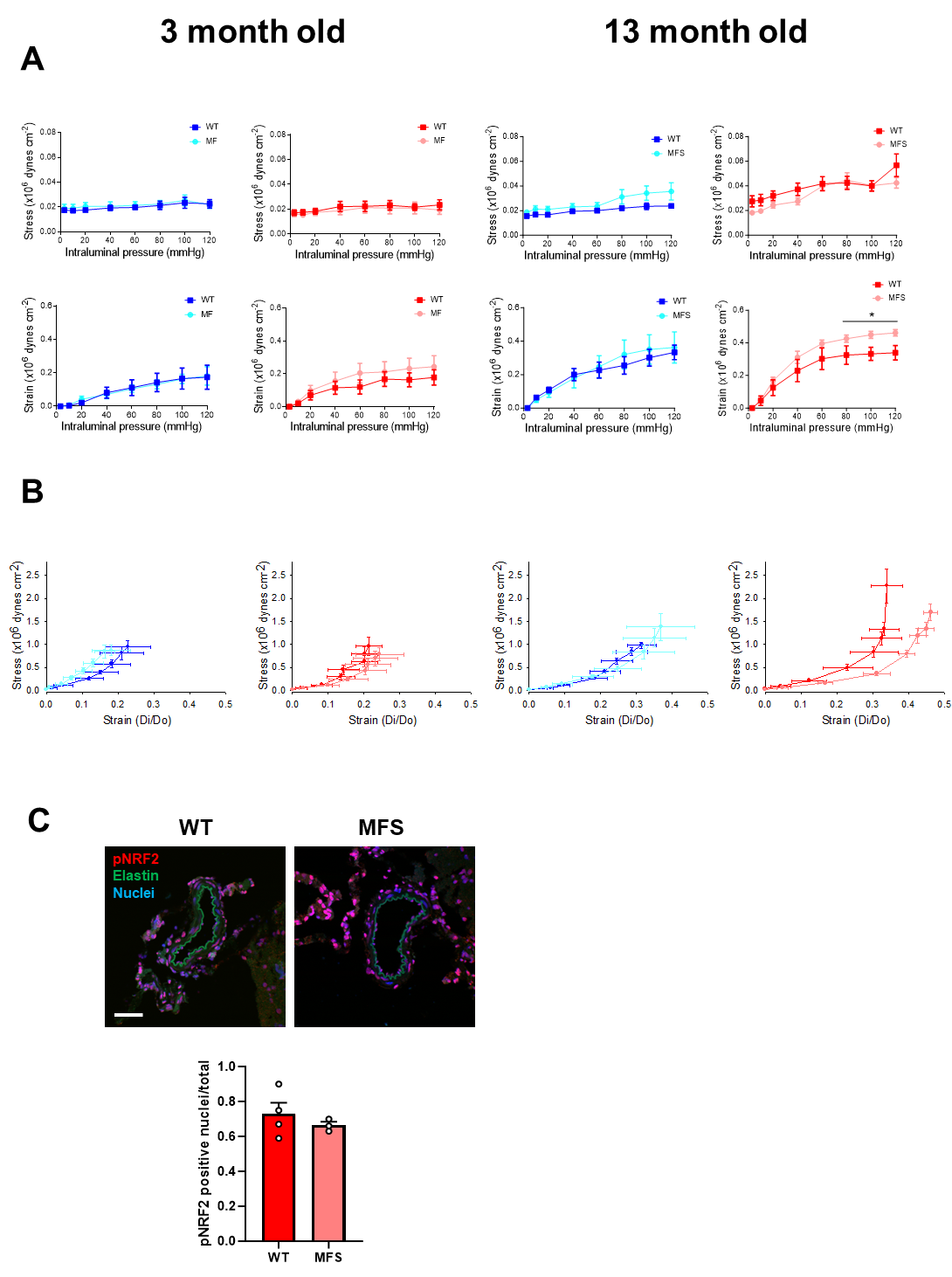


**Supplementary Figure 8.** Mechanical properties of basilar arteries from 3- and 13-month-old male and female wild-type (WT) and Marfan syndrome (MFS) mice and expression of phospho(p)-NRF2 in basilar arteries from 3-month-old female WT and MFS mice. (A) Stress-intraluminal pressure and strain-intraluminal pressure under passive conditions (0 Ca^2+^-Krebs-Henseleit solution). Results are the mean ± SEM of *n* = 7-8 (WT male), *n* = 5 (MFS male), *n* = 4-6 (WT female), and *n* = 5-8 (MFS female). **p* < 0.05 by repeated measures two-way ANOVA with Bonferroni's post-hoc test. (B) Stress-strain [observed internal diameter from a given intravascular pressure/internal diameter at 3 mmHg (Di/Do)]. Results are the mean ± SEM of *n* = 7-8 (WT male), *n* = 5 (MFS male), *n* = 4-6 (WT female), and *n* = 5-8 (MFS female). (C) Representative photomicrographs (top) and quantification (bottom) of pNRF2-positive nuclei (red immunofluorescence) relative to total nuclei (blue Hoechst 33342 staining) in confocal sections of basilar arteries from 3-month-old female WT and MFS mice. Natural autofluorescence of elastin (green) is also shown. Scale bar, 50 µm. Results are the mean ± SEM with each data point representing an animal.


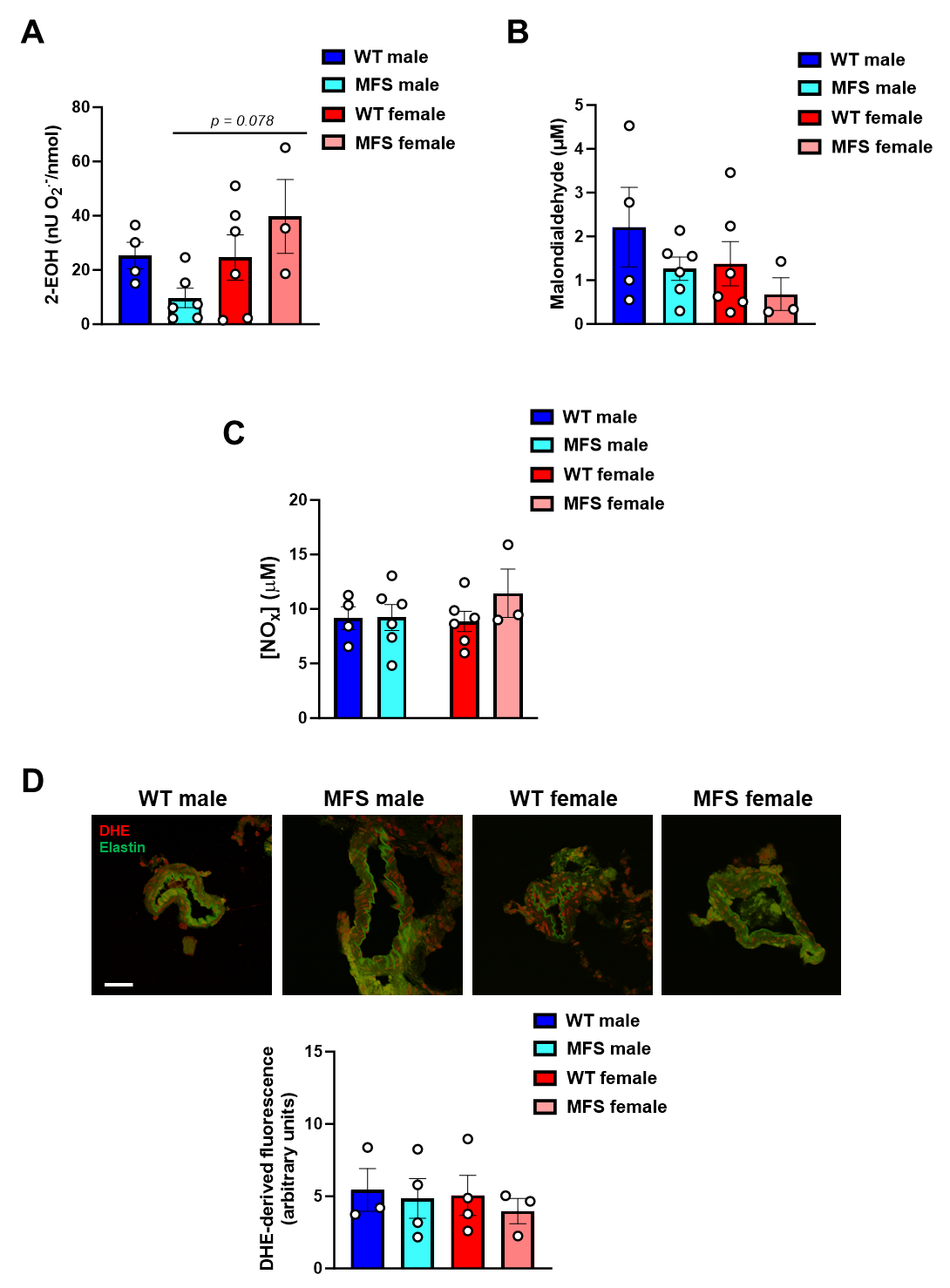


**Supplementary Figure 9.** Levels of some representative redox markers measured in 3-month-old male and female wild-type (WT) and Marfan syndrome (MFS) mice subjected to transient bilateral common carotid artery occlusion. (A) 2-hydroxyethidium (2-EOH) concentrations analyzed by HPLC, as an indirect indicator of superoxide anion. (B) Malondialdehyde concentrations, as an indirect indicator of lipid oxidation, measured by spectrophotometry. (C) Nitric oxide metabolites (nitrites and nitrates) concentrations measured by spectrophotometry. (D) Representative photomicrographs (top) and quantification (bottom) of fluorescence (red) intensity in confocal basilar artery sections labelled with the oxidative dye dihydroethidium (DHE). Natural autofluorescence of elastin (green) is also shown. Scale bar, 50 µm. Results are the mean ± SEM with each data point representing an animal. Statistical analysis was performed with two-way ANOVA with Tukey's post-hoc test.

**
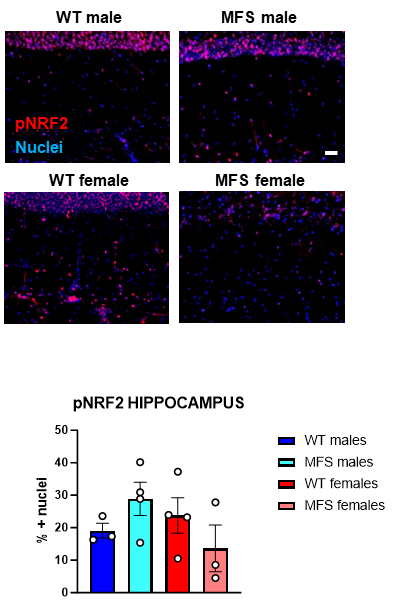
**

**Supplementary Figure 10.** Expression of phospho-NRF2 (pNRF2) in brains from 3-month-old male and female wild-type (WT) and Marfan syndrome (MFS) mice subjected to transient bilateral common carotid artery occlusion. Representative images of pNRF2 immunofluorescence (red) and nuclei staining (blue; DAPI) in brain hippocampus. Scale bar, 50 µm. Bar graphs show the results of the percentage of pNRF2-positive nuclei immunostaining in brain hippocampus. Results are the mean ± SEM with each data point representing an animal.
